# Multiple Cis-acting elements modulate programmed -1 ribosomal frameshifting in Pea enation mosaic virus

**DOI:** 10.1093/nar/gkv1241

**Published:** 2015-11-17

**Authors:** Feng Gao, Anne E. Simon

**Affiliations:** Department of Cell Biology and Molecular Genetics, University of Maryland College Park, College Park, MD 20742, USA

## Abstract

Programmed -1 ribosomal frameshifting (-1 PRF) is used by many positive-strand RNA viruses for translation of required products. Despite extensive studies, it remains unresolved how cis-elements just downstream of the recoding site promote a precise level of frameshifting. The Umbravirus *Pea enation mosaic virus* RNA2 expresses its RNA polymerase by -1 PRF of the 5′-proximal ORF (p33). Three hairpins located in the vicinity of the recoding site are phylogenetically conserved among Umbraviruses. The central Recoding Stimulatory Element (RSE), located downstream of the p33 termination codon, is a large hairpin with two asymmetric internal loops. Mutational analyses revealed that sequences throughout the RSE and the RSE lower stem (LS) structure are important for frameshifting. SHAPE probing of mutants indicated the presence of higher order structure, and sequences in the LS may also adapt an alternative conformation. Long-distance pairing between the RSE and a 3′ terminal hairpin was less critical when the LS structure was stabilized. A basal level of frameshifting occurring in the absence of the RSE increases to 72% of wild-type when a hairpin upstream of the slippery site is also deleted. These results suggest that suppression of frameshifting may be needed in the absence of an active RSE conformation.

## INTRODUCTION

The rapid, efficient life cycle of positive-strand RNA viruses requires optimal usage of their compact genomes for gene expression and RNA replication. A variety of non-canonical translation mechanisms have evolved in RNA viruses to allow protein production to be customized to specific needs at different stages of their life cycle (1–4). Programmed ribosomal frameshifting (PRF) and translational readthrough are two ribosome recoding mechanisms employed by a large number of plant-, fungal-, animal- and human-infecting viruses to control when translation of an ORF terminates at a stop codon or carries on to produce a C-terminally extended polypeptide (5–12). When -1PRF occurs, translating ribosomes shift one residue backward at a slippery sequence and then continue translation in the new -1 reading frame generating the extension product (13). In translational readthrough, the stop codon is decoded by a suppressor tRNA resulting in continued translation that generates the extended polypeptide (14). Since the recoded product of many animal and plant viruses is the viral RNA-dependent RNA polymerase (RdRp), maintaining a precise ratio of extension to termination products appears critical for efficient propagation of the virus within the host (6,8,15–17). Therefore, the recoding event must be strictly modulated during the viral life cycle.

Efficient eukaryotic -1PRF requires cis-acting frameshift signals typically composed of a heptameric slippery sequence and a Recoding Stimulatory Element (RSE) positioned just downstream from the slippery site. In eukaryotes, the slippery sequence is typically a series of seven nucleotides, X XXY YYZ (X is any nucleotide, Y is either A or U and Z is not G; 0 frame codons are underlined) that allows tRNAs in the A- and P-site of the translating ribosome to un-pair from 0 frame codons and re-pair with at least two of three residues in -1 frame codons during the frameshift event (2,10,18). The downstream RSE is often either an H-type pseudoknot, a stable imperfect hairpin, or a large bulged hairpin (13,18,19). Most models suggest that the RSE modulates -1PRF efficiency by inducing ribosomes to pause at the slippery sequence, thus affecting kinetics of the translating ribosomes (e.g. intrinsic unwinding activity) (10,20–22). The *human immunodeficiency virus* (HIV) RSE was also recently found to adopt an alternative conformation, suggesting that structural plasticity of RSE is also required to achieve proper levels of frameshifting *in vivo* (23). In addition to the slippery site and the RSE, other factors are important for modulating frameshift efficiency. For example changing the length of the spacer region reduces frameshifting of *Infectious bronchitis virus* (IBV), HIV and *human T-cell leukemia virus type II* (HTLV-2) RSEs in reporter constructs (24–26). Altering the nucleotides in the spacer region also has an impact on -1PRF in HIV and HTLV-2 (25).

The efficiency of -1PRF can also be modulated by trans-acting factors such as antisense oligonucleotides, miRNAs, proteins and antibiotics (27–30). Furthermore, there is increasing evidence that cis-acting elements external to the RSE region are important for proper levels of recoding. For example, -1PRF efficiency is affected by a short sequence immediately upstream of the slippery site in HIV and HTLV-2 (25). In addition, an upstream hairpin in *Severe acute respiratory syndrome* coronavirus (SARS-CoV) downregulates -1PRF in a reporter construct (31) and a Shine-Dalgarno sequence stimulates -1 frameshifting of the *Escherichia coli* dnaX gene via direct interactions with elongating ribosomes (32). For *Barley yellow dwarf virus* (BYDV; genus Luteovirus, family *Luteoviridae*), *Red clover necrotic mosaic virus* (RCNMV; genus Dianthovirus, family *Tombusviridae*) and likely other related viruses, the terminal loop of a 3′ terminal hairpin or other 3′ end proximal sequence participates in a long-distance base-pairing interaction with an RSE bulge loop, which is required for efficient -1PRF to synthesize the RdRp (33,34). Similar structures and interactions are also required for translational readthrough to express the RdRp in *Tobacco necrosis virus-D* (TNV-D; genus Betanecrovirus, family *Tombusviridae*), *Carnation Italian ring spot virus* (CIRV; genus Tombusvirus, family *Tombusviridae*) and *Turnip crinkle virus* (TCV; genus Carmovirus, family *Tombusviridae*) (6,35). It has been proposed that the long-distance interaction is involved in switching between the incompatible activities of RdRp translation and gRNA replication (34), but how the interaction contributes to stimulation of -1PRF/translational readthrough by an RSE remains unexplored. Additional examples of external elements modulating recoding include: (i) a short-distance base-pairing interaction between two stem-loops downstream of the slippery site that is required for -1PRF of bacterial transposable elements in the IS51 group of the IS3 family (36); and (ii) an intermolecular kissing loop-loop interaction in SARS-CoV that stimulates -1PRF through dimerization of the viral genomic (g)RNA (37). This growing number of critical base-pairing interactions between RSE and non-continuous elements suggests a general necessity for such interactions to precisely control recoding in gRNA. Since the vast majority of studies on ribosome recoding make use of reporter constructs into which limited sequences from the recoding region are inserted, many of these critical interactions have likely been missed, leading to an incomplete and possibly misleading picture of the complete recoding process.

*Pea enation mosaic virus* (PEMV) consists of two positive-sense, single-stranded viral RNAs, the Luteovirus-related PEMV RNA1 and Umbravirus PEMV RNA2 (38) [Umbraviruses have not been assigned to a virus family; however, the 5′ half of Umbraviruses is related to viruses in the *Tombusviridae*]. Whereas both viral RNAs code for their own RdRp and can replicate independently in cells, PEMV RNA2 depends on the encoded coat protein of PEMV RNA1 for encapsidation and transmission (39). The 4252 nt genome of PEMV RNA2 (referred to as PEMV in this report), which lacks a 5′ cap structure and a 3′ poly(A) tail (39), contains multiple essential cap-independent translation enhancers in the 3′ UTR that are required for efficient translation of viral proteins (40–43). Among its four open reading frames (ORFs), the RdRp (p94) is translated from the gRNA via -1PRF of 5′-proximal ORF1, which codes for p33 (39). The movement proteins (p26 and p27) are encoded by two overlapping ORFs (ORF3 and ORF4) and are expressed from at least one subgenomic (sg)RNA during infection (44,45).

For this study, we investigated a variety of factors that influence -1PRF using full-length PEMV gRNA, with particular focus on the roles of cis-acting RNA elements outside of the RSE. The PEMV RSE is a large hairpin with two internal asymmetric bulges immediately downstream from a non-canonical slippery site. Mutational analysis of the RSE revealed the importance of sequences and structures for efficient activity, and also suggested that (at least) the lower stem can adopt an alternate structure. Long-distance base-pairing with a 3′ terminal hairpin was necessary for efficient frameshifting, but was less important when the RSE lower stem was stabilized. The length and sequence of the spacer region between the RSE and the slippery site, as well as the stop codon after the slippery site, contributed to frameshifting efficiency. Surprisingly, the slippery sequence in the absence of the RSE mediated a basal level of frameshifting that increased to 72% of wild-type (WT) levels when an upstream, phylogenetically conserved hairpin was eliminated. This suggests that hairpins or other elements upstream of slippery sites may be needed to suppress frameshifting if the RSE adopts an inactive alternative conformation.

## MATERIALS AND METHODS

### Construction of PEMV mutants

Plasmid pUC19-PEMV, which contains the full-length wild type PEMV RNA2 genome downstream of a T7 promoter, was used as a template for polymerase chain reaction (PCR)-based site-directed mutagenesis. The desired mutations were introduced using custom designed oligonucleotide primers (Integrated DNA Technologies) using the QuikChange one-step site-directed mutagenesis procedure (46). The resulting PCR products were subjected to DpnI digestion before introduction into competent DH5α *E. coli* cells. The presence of the desired mutations was confirmed by sequencing (Eurofins Genomics).

### *In vitro* transcription and translation

Uncapped PEMV WT and mutant gRNA transcripts were generated by *in vitro* transcription using bacteriophage T7 RNA polymerase and SmaI-linearized pUC19-PEMV DNA plasmids. One pmol of *in vitro* synthesized transcripts was translated in 10 μl of wheat germ extracts (WGE) (Promega) in the presence of ^35^S-methionine according to the manufacturer's instructions, with the addition of 100 mM potassium acetate. After incubation at 25°C for 1.5 h, the reaction mixture was resolved on a 10% SDS-PAGE gel. The gel was dried and exposed to a phosphorimager screen, which was subsequently scanned by a FLA-5100 fluorescent image analyser (Fujifilm). The intensity of radioactive bands was quantified using Quantity One software (Bio-Rad).

### Protoplast transfection and viral RNA detection

Arabidopsis callus culture protoplasts were prepared and transfected using a polyethylene glycol-mediated transformation protocol as previously described (42). Briefly, 20 μg of PEMV gRNA transcripts were transfected into 5 × 10^6^ protoplasts and incubated at 22°C for 24 h in the dark. Total RNA was extracted using RNA extraction buffer (50 mM Tris-HCl [pH 7.5], 5 mM EDTA [pH 8.0], 100 mM NaCl, 1% SDS), followed by phenol-chloroform extraction and ethanol precipitation. Five micrograms of total RNA was subjected to electrophoresis through a 1.5% non-denaturing agarose gel and then denatured and transferred to nylon membrane by capillary transfer. [α-^32^P] dCTP labeled DNA probes complementary to the 3′UTR of the genome were used for hybridization. The blot was exposed to a phosphorimager screen, which was scanned by a FLA-5100 fluorescent image analyzer (Fujifilm). The viral gRNA accumulation was quantified using Quantity One software (Bio-Rad).

### SHAPE structural probing

SHAPE structural probing was performed essentially as previously described (40). Briefly, PEMV gRNA was denatured at 95°C for 3 min, snap-cooled on ice for 2 min and then incubated in SHAPE folding buffer (80 mM Tris-Cl, [pH 8.0], 11 mM Mg(CH_3_COO)_2_, 160 mM NH_4_Cl) at 37°C for 20 min. Folded RNA was then treated either with 15 mM N-methylisatoic anhydride (NMIA) or with the same volume of dimethyl sulfoxide (DMSO) as a negative control at 37°C for 40 min. RNA was recovered by ethanol precipitation and then re-suspended in 0.5x TE buffer. Primer extension reactions were performed using [γ-^32^P] ATP-labeled oligonucleotides and SuperScript III reverse transcriptase (Invitrogen) as previously described (47). Primers complementary to PEMV positions 1052–1077 (5′-CCAAAATCTCCAAGAGGACGCACAAC-3′) and 1165–1185 (5′-GAGGGGAAGGGAACCTTAGGC-3′) were used for structural probing of the RSE/SLA and SLB, respectively. Reaction products along with ladders generated by Sanger sequencing were resolved on 8% urea-based polyacrylamide gels. Gels were then dried and exposed to a phosphorimager screen. The NMIA reactivity of each nucleotide was assigned none, low to moderate and moderately high to high by visually inspecting the intensities of individual bands. RNA secondary structures were generated from structure probing results and the best-fitting Mfold predictions (48).

## RESULTS

### Analysis of the frameshift motif of PEMV

**Figure 1.**
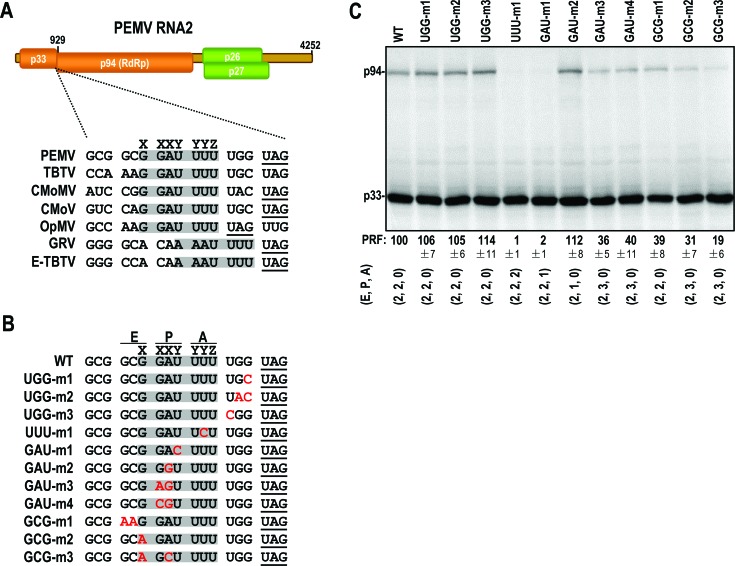
Role of E, P and A slippery sequence codons on -1PRF efficiency in WGE. (**A**) Top: genome organization of PEMV RNA2. p33 is likely a replication-associated protein based on other viruses in the *Tombusviridae*. The p94 RdRp is expressed from a -1PRF event just before the termination of p33. p26 and p27 are expressed from sgRNA(s); bottom: comparison of Umbravirus sequences immediately upstream of their first ORF stop codon (UAG, underlined). *Tobacco bushy top virus* (TBTV) (KM 067277.1); *Carrot mottle mimic virus* (CMoMV) (NC_001726.1); *Carrot mottle virus* (CMoV) (NC_011515.1); Opium poppy mosaic virus (OpMV) (EU151723); *Groundnut rosette virus* (GRV) (NC_003603.1); *Ethiopian tobacco bushy top virus* (E-TBTV) (NC_024808). Predicted slippery sequences are shaded. ‘X XXY YYZ’ denotes the motif of conventional slippery sequences. (**B**) Alterations (in red) generated in or near the slippery sequence. Codon positions within a paused ribosome before frameshifting are denoted as E, P or A. (**C**) *In vitro* translation of WT and mutant gRNAs. Number of codon:anticodon mismatches in the E-, P- and A-sites when frameshifting occurs is indicated. Each non-Watson–Crick base-pair (includes non-third position G:U) are given a value of ‘1’.

In PEMV, the slippery sequence ‘5′-G GAU UUU-3′’ is located 3-nt upstream of the p33 amber stop codon (Figure 1A). This sequence does not follow the conventional heptameric slippery sequence X XXY YYZ as only the A-site tRNA^AAA^ can perfectly re-pair with the -1 frame codon during the frameshifting event. The same slippery sequence is found in four other Umbraviruses at the same position relative to the RSE. The exceptions, *Groundnut rosette virus* (GRV) and *Ethiopian tobacco bushy top virus* (E-TBTV), have a conventional heptameric slippery sequence (AAAUUUU) just upstream of the stop codon (Figure 1A). To investigate the role of nucleotides within and flanking the slippery sequence on -1PRF, mutations were introduced into full-length PEMV gRNA (Figure 1B) and levels of p94 were assayed for in WGE (Figure 1C). gRNA containing mutations in the variable ‘GG’ just upstream of the p33 stop codon as well as in the flanking conserved ‘U’ had near WT levels of frameshifting (UGG-m1, UGG-m2 and UGG-m3). Mutations that targeted the four uridylates upstream of the UGG (UUU-m1 and GAU-m1) reduced -1PRF to near background levels. Changing the PEMV slippery sequence (GGAUUUU) to the BYDV slippery sequence (GGGUUUU; GAU-m2), which allows the P-site tRNA^CCA^ to form an additional base-pair in the -1 frame, produced 112% of WT frameshifting. In contrast, prohibiting the re-pairing of the P-site tRNA (GAU-m3 and GAU-m4) reduced frameshifting to 36% and 40% respectively, suggesting that re-pairing of the P-site tRNA has a moderate impact on -1PRF efficiency.

Since it was suggested that the E-site tRNA is involved in -1PRF of HIV-1 (49), two mutations were generated in the first two positions (GCG-m1) or the third position (GCG-m2) of the E-site codon. These mutations reduced -1PRF to 39% and 31%, respectively (Figure 1C). Addition of a P-site codon mutation into the GCG-m2 background (GCG-m3) further reduced -1PRF to 19%, suggesting an additive effect from these particular alterations of the E- and P-site codons.

To examine if a correlation exists between -1PRF efficiency and the number of codon-anticodon base-pair mismatches encountered by tRNAs during frameshifting in WT and mutant gRNAs, the number of mismatches for the frameshifted E-site tRNA, P-site tRNA and A-site tRNA was annotated. Non-canonical base-pairs are considered to be mismatches and were given a score of 1 and G:U wobble base-pairs are not penalized. As illustrated in Figure 1C, a negative correlation exists between frameshift efficiency and mismatches at the P-site and A-site, with higher penalties for A-site mismatches. In contrast, there was no correlation between frameshifting and mismatches in the E-site. However, there was an impact of the E-site codons on frameshifting related to the identity of the residues (compare GCG-m1 with WT; Figure 1C).

### A large bulged stem-loop structure immediately after the stop codon modulates -1PRF in PEMV

**Figure 2.**
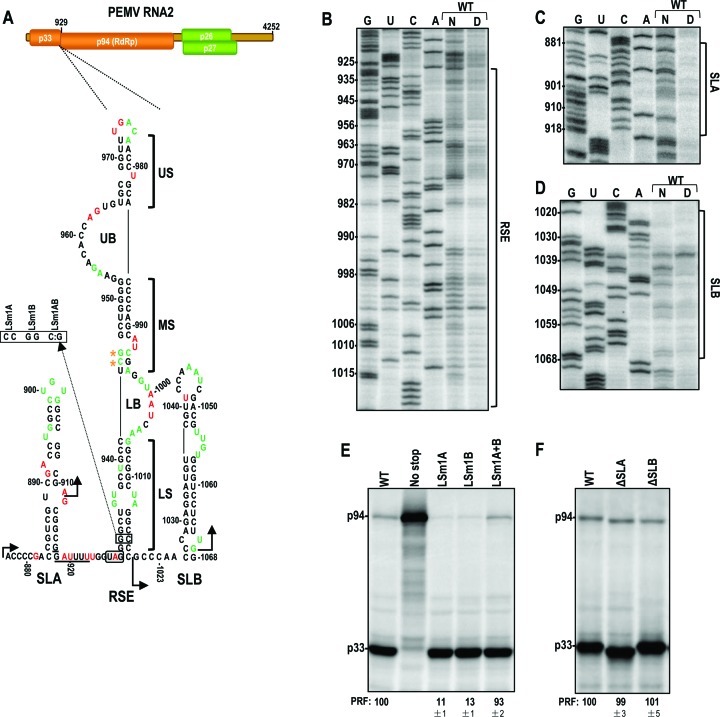
Identification of the RSE that modulates -1 PRF in PEMV RNA2. (**A**). Phylogenetically conserved hairpins near the p33 stop codon. Red residues, moderately-high to high reactivity to NMIA; green residues, low to moderate reactivity. Orange asterisks denote residues with variable SHAPE data in multiple repetitions (either no or low reactivity). Arrows denote deletion endpoints for constructs ΔSLA and ΔSLB. LS: lower stem; LB: lower bulge; MS: middle stem; UB: upper bulge; US: upper stem. The slippery sequence is underlined. p33 UAG stop codon is shaded. (**B**) Typical SHAPE phosphorimages for the RSE, (**C**) SLA or (**D**) SLB regions. Lanes G, U, C and A are ladder lanes. N, NMIA-modified; D, DMSO-treated controls. Positions of selected guanylates are shown at the left. The corresponding regions are shown at the right. (**E**) *In vitro* translation in WGE of WT and mutant gRNAs. Positions of p33 and p94 are shown. No stop, base insertion in the stop codon fusing the 0 and -1 frames; LSm1A, LSm1B and LSm1AB alterations are shown in (A). PRF, relative -1 PRF efficiency, calculated by normalizing the p94/p33 ratio compared with WT. Mean values and standard error throughout this study were calculated from at least three independent experiments. (**F**) *In vitro* translation of PEMV containing deletions of SLA (ΔSLA) or SLB (ΔSLB).

To identify the RSE that stimulates -1PRF in PEMV, sequences surrounding the p33 stop codon were analyzed by mFold (48), and full length gRNA was also subjected to RNA structure probing using selective 2′-hydroxyl acylation analyzed by primer extension (SHAPE). SHAPE reports on covalent linkage of NMIA to the 2′OH of flexible bases, which impedes reverse transcriptase-mediated primer extension. Typical SHAPE phosphorimages for this region are shown in Figure 2B–D. The RNA secondary structure that best fits the SHAPE data is shown in Figure 2A. Residues with moderately-high to high reactivity with NMIA are colored red and residues with low to moderate reactivity are colored green. The SHAPE data are mainly consistent with three hairpins: a large central hairpin with two internal asymmetric bulges (UB, upper bulge and LB, lower bulge), and two smaller hairpins (SLA and SLB) flanking the central hairpin. U943 is arbitrarily paired with A996 in the central hairpin middle stem (MS), although alternative pairing of U943 with A1005 in the lower stem (LS) is also possible. While the proposed structure is a good fit for most of the SHAPE data, several elements were not consistent with the NMIA reactivity profile. In particular, most UB residues were not susceptible to NMIA modification suggesting that this region in the gRNA is likely not single-stranded as depicted in the figure. In addition, the lower portion of the MS consistently contained moderately flexible residues C994 and A996, and about half of the SHAPE gels contained flexible partner residues (orange asterisks, Figure 2A). The flexibility of this region of the MS suggests either that this portion of the stem is not stable, or that these residues may be adopting an alternative conformation in some percentage of the gRNAs in the population.

Six additional Umbraviruses have full-length sequences deposited in Genbank (*Tobacco bushy top virus*,TBTV; *Carrot mottle mimic virus*, CMoMV; *Carrot mottle virus*, CMoV; GRV; E-TBTV and *Opium poppy mosaic virus*, OpMV). All of these viruses are predicted to have a similar arrangement of three hairpins in the analogous location in relation to their slippery sites (Supplementary Figure S1, A–F). The central hairpins of all Umbraviruses are of similar lengths, ranging from 88 to 95 nt, and all are 2 to 5 nt downstream of their slippery sites. The base stems of these central hairpins begin with four or five G:C pairs, with the 5′ guanylate comprising the G of the UAG termination codon in five of the seven gRNAs, including PEMV. Six of seven of these hairpins also have a second stretch of the same number of G:C base-pairs in their MS. All RSE have two large internal bulge loops with the exception of CMoV, which is predicted to have the lower of the two bulge loops along with an oversized terminal loop.

To determine if the three hairpins in the vicinity of the slippery site are important for frameshifting in PEMV, mutations that disrupt and restore the LS of the central hairpin were engineered in the gRNA (LSm1A, LSm1B and LSm1AB; Figure 2A). Since these mutations (and all other possible LS base-pair alterations) change the primary sequence of the RdRp, mutant gRNAs were only subjected to *in vitro* translation in WGE, with the amount of p94 synthesized correlating with the efficiency of -1PRF. LSm1A with a G:C to C C disruption or LSm1B with a G:C to G G disruption reduced synthesis of p94 by 89% and 87%, respectively (Figure 2E). Combining both mutations (LSm1AB), which should re-establish LS base-pairing, restored p94 levels to 93% of WT. To determine if flanking hairpins SLA and SLB are also important for -1PRF in the gRNA *in vitro*, gRNA containing deletions of either SLA or SLB (ΔSLA, ΔSLB) were subjected to translation in WGE. Neither deletion affected the efficiency of frameshifting (Figure 2F), suggesting that despite being phylogenetically conserved throughout the *Tombusviridae* (SLA) or in Umbraviruses (SLB), they are not participating in a discernable role in this assay. Since these results suggest an important role for the central hairpin just downstream from the slippery site in -1PRF, this hairpin was designated an RSE.

### Both sequence and structure are important for PEMV RSE function

To determine the importance of various PEMV RSE sequences and secondary structures for -1PRF, alterations were generated throughout the RSE in full-length gRNA, and synthesis of p94 was assayed for in WGE. gRNA containing silent mutations that do not alter the amino acid sequence of the RdRp were also inoculated into Arabidopsis thaliana protoplasts and levels of accumulating gRNA were determined at 24-hour post-inoculation (hpi) using RNA gel blots.

**Figure 3.**
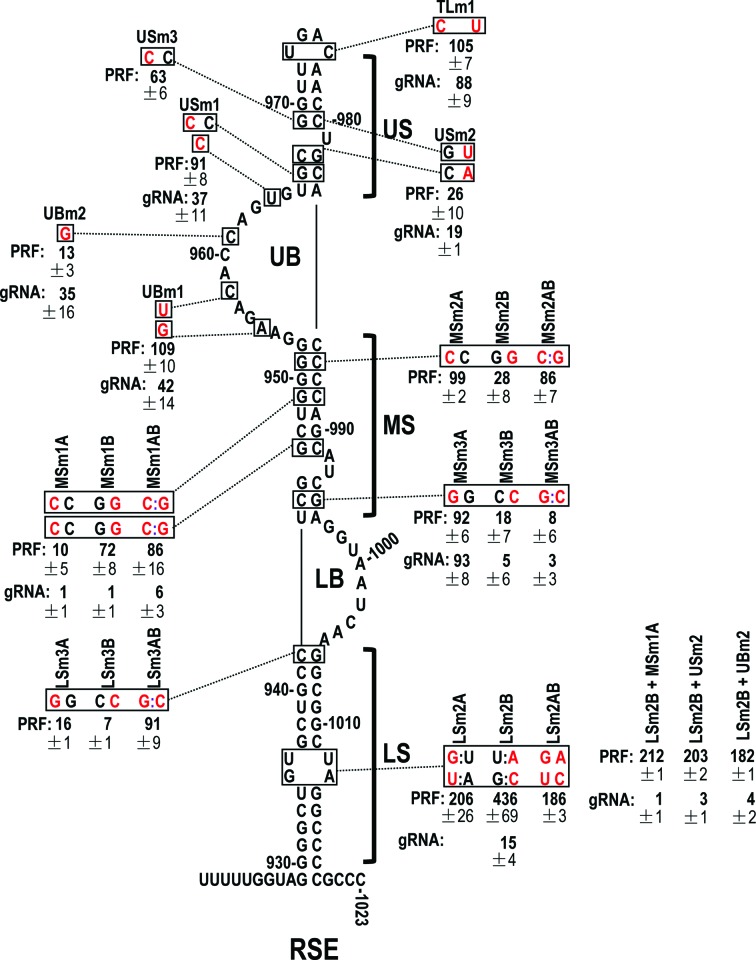
Mutational analysis of the RSE. Relative -1PRF efficiency was determined by *in vitro* translation assays and normalized to WT (see legend to Figure 2); gRNA, relative viral gRNA accumulation in protoplasts at 24 hpi as determined by RNA gel blot analysis. Density of viral gRNA bands was normalized to rRNA loading controls and values expressed as a percentage of WT. Only mutants harboring silent mutations were analyzed in protoplasts. Base alterations are in red.

To investigate if the two-base symmetrical loop in the LS contributes to frameshifting, two sets of mutations were generated that closed the loop by converting the U U/G A to G:U/U:A or U:A/G:C. The presence of G:U/U:A (LSm2A) or U:A/G:C (LSm2B) increased -1PRF activity by 2- and 4-fold, respectively (Figure 3). Changing the loop residues without canonical base pairing by incorporating both sets of mutations (G A /U C) also enhanced -1PRF by 1.9-fold. These data suggest a positive correlation between the stability of the LS in the RSE and -1PRF efficiency, but also that the identity of residues in the small loop can contribute toward -1PRF efficiency, possibly by non-canonical pairing across the stem. To determine if enhanced levels of p94 affect gRNA accumulation, WT gRNA and LSm2B were inoculated into protoplasts and levels were compared at 24 hpi. LSm2B accumulated to only 15% of WT PEMV, suggesting that enhanced RdRp synthesis (or other consequence of these mutations) does not correlate with enhanced gRNA accumulation in single cells *in vivo*.

Single mutations in the top base pair of the LS (LSm3A, LSm3B) reduced p94 levels by 84% and 93%, respectively. LSm3AB, which combined both mutations and should reform the putative base-pair, restored -1PRF to 91% of WT (Figure 3). This result supports the pairing of C942 with G1006, and not G997, which was an alternative possibility. In contrast, mutations designed to disrupt and reform the middle stem (MS) did not give results with a simple interpretation. MSm3A (C944G), which disrupts the putative pairing of C944 with G995 in the lower MS, had little effect on levels of p94. However, alteration of G995 (MSm3B) reduced p94 levels to 18% of WT, and levels were not restored when both mutations were combined (MSm3AB). Similar results were obtained when gRNA containing these silent mutations were assayed for accumulation in protoplasts (Figure 3). These results suggest that G995 is critical for -1PRF, and a base pair in this location is either not forming or is not important. Disruption of two base pairs in the upper MS (G946C and G949C; MSm1A) reduced p94 levels by 90%. However, altering the two putative partner residues on the 3′ side of the stem (C988G and C991G; MSm1B) was less detrimental, reducing -1PRF by just 28%. gRNA containing both sets of mutations had only a minimal effect on p94 levels (86% of WT), suggesting that the combined alterations mitigated the effects of G946C and G949C alone. All three combinations of mutations (all of which are silent mutations) reduced gRNA levels in protoplasts to near undetectable levels, suggesting that maintaining the WT sequence in these locations is critical *in vivo*. Similar results were obtained for disrupting a putative base-pair in the MS just below the UB. Alteration of G951C (MSm2A) had no discernable effect on -1PRF, whereas altering the putative partner residue C986G (MSm2B) reduced p94 levels to 28% of WT. Combining both mutations partially corrected the defect due to C986G, restoring p94 levels to 86% of WT.

The combination of G967C, which is normally paired with C983 in the US, and UB residue alteration U964C (USm1), had little effect on -1PRF (91% of WT) but was detrimental for gRNA accumulation *in vivo* (37% of WT). Other mutations that disrupted base-pairing in the US (USm2 and USm3) reduced -1PRF to 26% and 63% of WT, respectively. In addition, USm2, which changed C:G and G:C pairs to G:U and CA, reduced gRNA levels to 19% of WT. In contrast, altering the terminal loop (TLm1) did not negatively affect -1PRF and had only a marginal effect (88% of WT) on gRNA accumulation *in vivo*. These results suggest that the US is important but not critical for frameshifting.

Additional silent mutations were also generated in the UB. Alteration of A955G and C958U (UBm1) had no detrimental effect on -1PRF but did reduce gRNA accumulation to 42% of WT. C961G (UBm2) reduced p94 levels to 13% of WT and gRNA accumulation to 35% of WT. In total, these results suggest that base-pairing in the LS, and WT sequence in a portion of the UB, are critical for -1PRF. In addition, although the MS stem is not necessary for efficient -1PRF *in vitro* frameshifting, several residues in this location are needed for functionality of the RSE.

We also determined whether enhancing the stability of the LS can mitigate the negative effect of mutations in the upper portion of the RSE. LSm2B, which enhanced p94 synthesis by 4-fold, was combined with MSm1A (10% of WT), USm2 (26% of WT) and UBm2 (13% of WT). For all three combinations, -1PRF of LSm2B was reduced by approximately 50% (Figure 3). This suggests that enhancing the stability of the LS partially mitigates the negative effects of upper RSE mutations. However, gRNAs containing the combined (all silent) mutations accumulated at near background levels, suggesting that PEMV frameshifting *in vivo* is likely a complex process requiring many regions of the RSE.

### SHAPE structural probing of RSE mutants

**Figure 4.**
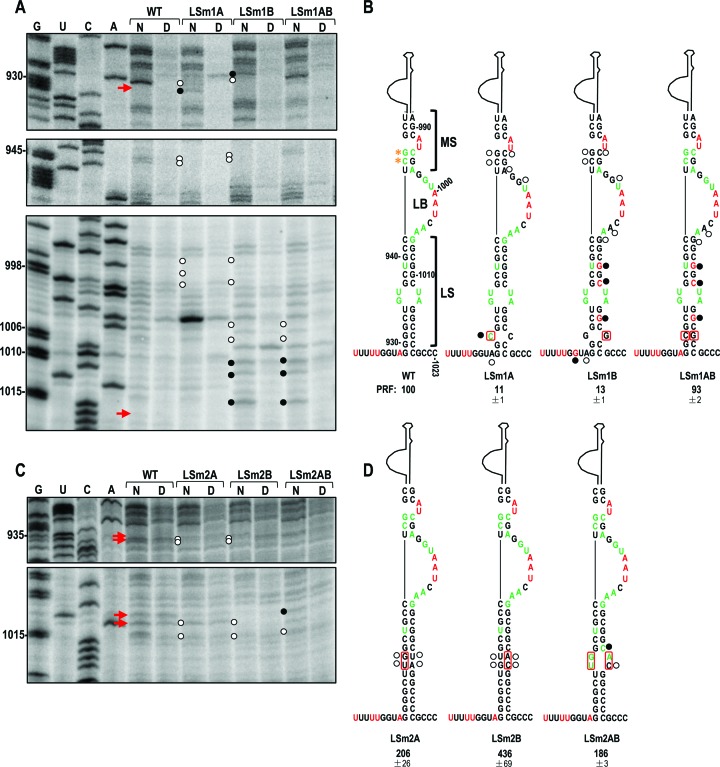
Effect of LS mutations on the structure of the RSE. (**A**) SHAPE phosphorimages showing the effect of mutations LSm1A, LSm1B and LSm1AB on the structure of the RSE. Only regions with residue reactivity changes are shown. The positions of base alterations are denoted by red arrows. (**B**) Secondary structures of WT, LSm1A, LSm1B and LSm1AB RSE. Location of the mutations are boxed in red. Filled and open circles denote residues with increased or reduced reactivity to NMIA, respectively, compared to WT. Only changes that were consistent in independent experiments are denoted. G, U, C and A, nucleotide ladder lanes; N, NMIA treated; D, DMSO treated control. Positions of selected guanylates are shown at the left. Residue coloring and meaning of filled and open circles is described in legend to Figure 2. Orange asterisks denote residues with variable SHAPE data in multiple repetitions (no or low reactivity). Relative -1PRF values are from Figure 2. (**C**) SHAPE phosphorimages showing the effect of mutations in LSm2A, LSm2B and LSm2AB. (**D**) WT RSE secondary structure and proposed secondary structures for LSm2A, LSm2B and LSm2AB. Relative -1PRF values are from Figure 3.

To better understand the results of the RSE genetic analysis, SHAPE structure probing was performed on full-length PEMV gRNA containing selected mutations. LSm1A (G931C), which is located near the base of the LS and reduced -1PRF by nearly 9-fold (Figure 2), enhanced the flexibility of the mutated residue (Figure 4A and B, filled circle), but surprisingly did not alter the flexibility of its partner residue (C1017). In addition, all residues in the lower portion of the MS that had been consistently or occasionally flexible were no longer susceptible to NMIA (C944, G945, C994, A996 and G998) (Figure 4A and B, open circles), suggesting that G931C is stabilizing this portion of the MS. Furthermore, normally highly reactive residue A928 (the adenylate in the UAG stop codon upstream of the RSE) became inflexible. These consequences of G931C suggest: 1) higher order structure in the RSE connects the LS, MS, and adjacent sequence upstream of the RSE; and 2) the possibility that the base of the LS is not forming in the gRNA synthesized *in vitro*. The LSm1A partner residue, LSm1B (C1017G), which also reduced frameshifting by nearly 7-fold, caused no local structural changes. Instead, three residues on only the 3′ side of the LS (the same side as C1017) became highly reactive, and two residues in the LS and LB lost their reactivity. Similar to LSm1A, residues upstream of the RSE, including A928, showed altered reactivity. Also similar to LSm1A, the lower stem of the MS contained fewer flexible residues. In the compensatory mutant LSm1AB, the structural changes observed for LSm1B in the LS and LB were retained, whereas the MS residues and upstream sequence flanking the RSE regained WT flexibility. For both LSm1B and LSm1AB, no detectable differences were discernable in the 5′ side of the LS. These results support a connection among sequence flanking the RSE, the LS, and the MS in the 3-D structure of the RSE, and do not support the structure of the LS shown in the figure. However, since LSm1A and LSm1B were genetically compensatory in WGE, and since the LS is phylogenetically conserved in all known or putative RSE structures throughout the *Tombusviridae* (see discussion), it seems likely that, at least *in vitro*, more than one conformation exists for the PEMV RSE.

LSm2A (GU935UG), designed to strengthen the LS by closing the small symmetrical bulge, reduced the flexibility of residues on both sides of the bulge, suggesting that U:A and G:U base pairs are forming (Figure 4C and D). Similar structural changes were observed for LSm2B, which was also designed to stabilize the LS. Combining the mutations in LSm2AB, which should re-establish the small bulge, restored flexibility to most bulge residues. In addition, residue C1011 flanking the loop became more flexible. Since no changes were discernable elsewhere in the RSE or upstream sequence, these data suggest that the internal bulge can be closed without disturbing the putative interactions between the LS, MS and upstream sequence.

**Figure 5.**
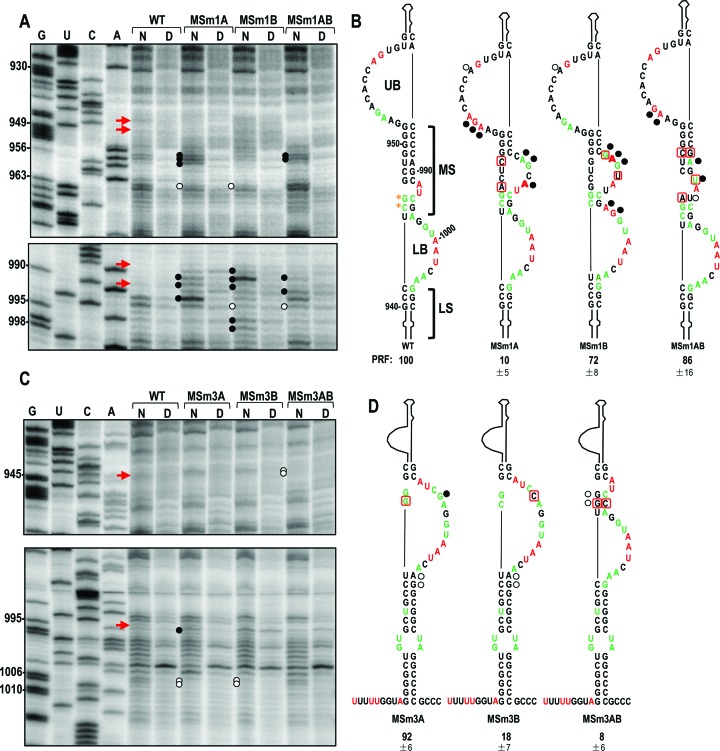
Effect of MS mutations on structure of RSE. (**A**) SHAPE phosphorimages and (**B**) secondary structures of WT, MSm1A, MSm1B and MSm1AB. (**C**) SHAPE phosphorimages and (**D**) proposed secondary structures of MSm3A, MSm3B and MSm3AB. See legend to Figure 4 for more information.

Two sets of mutations were assayed that target the MS. MSm1A, with two alterations on the 5′ side of the MS, caused substantial changes in residue flexibility on the 3′ side of the MS, as well as enhancing flexibility of three residues in the UB (Figure 5A and B). Disruption of the MS by base alterations on the 3′ side of the MS caused similar flexibility changes on the 3′ side of the stem, with additional flexibility changes extending down into the LB. In the UB, 955AGA retained WT flexibility, whereas A962 lost NMIA reactivity, as was also found for MSm1A. This suggests that flexibility changes in most of these UB residues were related to the specific base alterations in MSm1A and not to a general disruption of the MS. The SHAPE profile for the compensatory mutant MSm1AB support this suggestion, as several of the UB flexibility changes remained. A962, however regained flexibility, suggesting that flexibility of this residue is connected with the structure of the MS. Although MSm1AB reduced changes in residue flexibility on the 3′ side of the MS, some residues remained reactive to NMIA, suggesting that the MS structure differs from WT in at least some molecules within the population.

The lower MS in the WT gRNA contains several residues that are moderately susceptible to NMIA and several that are not consistently flexible. In addition, as described above, alterations in the lower LS caused these MS residues to lose flexibility, suggesting that base-pairing across this portion of the MS stem was strengthening. To investigate the effect of mutations in this MS region on the flexibility profile of the RSE, the MSm3 set of single and compensatory mutations were subjected to SHAPE. MSm3A (C944G) slightly enhanced the flexibility of its pairing partner G995 in MSm3A (Figure 5C and D). In addition, A1005 and G1006 lost flexibility suggesting that U943 and A1005 were now pairing. None of these changes affected frameshifting, which remained near WT levels. MSm3B (G995C), which caused a 5-fold drop in -1PRF, did not discernably impact the flexibility of C944, but did cause the same reduced flexibility for A1005 and G1006. Compensatory mutation MSm3AB reduced flexibility of residues on the 5′ side of the lower MS stem and restored WT flexibility to A1005 and G1006, suggesting weak formation of the lower MS. Although the two mutant residues (G944:C995) were predicted to pair in MSm3 AB, -1PRF remained inefficient. As described in the next section, G995 is also involved in communication with a distal element that is required for efficient frameshifting. These results demonstrate that disruption of the lower MS does not lead to reciprocal structural alterations in the lower LS stem (see Figure 4B).

### Long-distance base-pairing interaction between the RSE and a distal element modulates -1PRF

**Figure 6.**
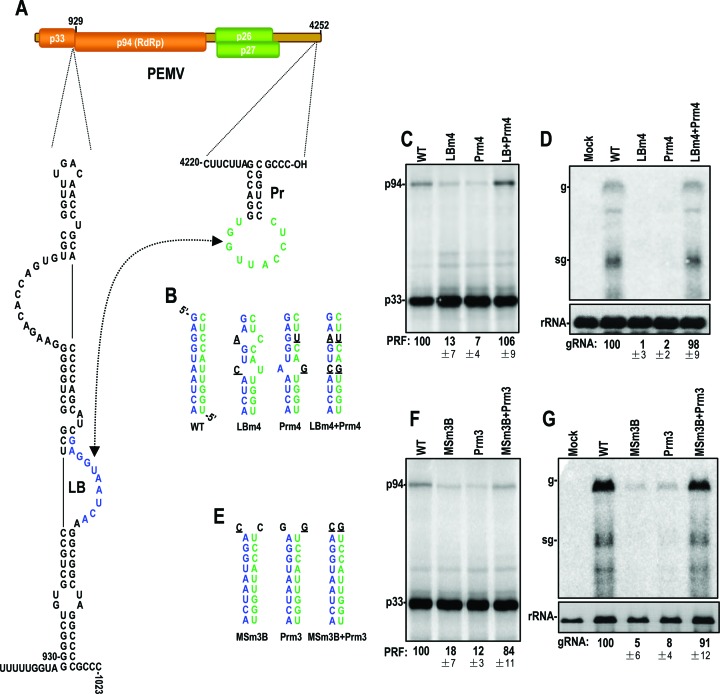
Long-distance RNA:RNA interaction between the RSE LB and 3′ proximal Pr loop is required for efficient -1PRF. (**A**) Long-distance interaction between LB (blue) and Pr loop (green). (**B**) Base alterations are colored black and underlined. (**C**) *In vitro* translation of WT and mutant gRNAs. (**D**) RNA gel blot analysis of PEMV gRNA levels in Arabidopsis protoplasts at 24 hpi. Positions of gRNA (g) and sgRNA (sg) are indicated. 28S ribosomal RNA (rRNA) served as the loading control. (**E**) Base alterations in mutants MSm3B, Prm3 and MSm3B+Prm3. (**F**) *In vitro* translation of WT and mutant gRNAs. (**G**) RNA gel blot analysis of WT and mutant gRNA.

In BYDV and RCNMV, a distal stem-loop structure near the 3′ end of the genome modulates -1PRF through a long-distance base-pairing interaction with an RSE asymmetric bulge loop (33,34). Similar interactions with 3′ sequences are known or predicted to be important for translational readthrough leading to RdRp synthesis throughout the *Tombusviridae* (6,35). The 3′ terminal hairpin of PEMV gRNA, known as the Pr (40), harbors a 10-nt loop sequence (3′-CUCCAUUGGU-5′) that is complementary to the lower bulge (LB) of the RSE (5′-GAGGUAAUCA-3′) (Figure 6A). Similarly positioned long-distance interactions are also possible for all Umbraviruses (Supplementary Figure S2). To determine the importance of the proposed interaction for frameshifting in PEMV, several sets of mutations were introduced into the PEMV gRNA to disrupt or restore putative complementarity between the RSE and Pr loop. Altering an adenylate in the RSE to U (5′-GAGGUUAUCA-3′) reduced frameshifting by 12-fold, however mutation of the Pr partner residue to A (3′-CUCCAAUGGU-5′) only reduced frameshifting by 9% (Supplementary Figure S3C). The two mutations together, which should re-establish pairing, improved frameshifting to WT levels, suggesting that the Pr alteration might still support the long-distance pairing. Similar results were obtained for accumulation of mutant gRNA in protoplasts (Supplementary Figure S3D). A second set of mutations in the same position (A:U to G:U or A C) reduced frameshifting significantly only for the A C mismatch, with the compensatory change A:U to G:C restoring frameshifting to WT levels (Supplementary Figure S3C). None of these latter mutants accumulated efficiently in protoplasts, suggesting that the 3′ Pr alteration may have an adverse effect on replication (Supplementary Figure S3D). Since these results were not definitive, an additional set of mutations were generated that targeted two base-pairs simultaneously (LBm4, Prm4; Figure 6B). Mutations that disrupted the base-pairing reduced p94 levels by 8- to 14-fold, whereas mutations that were designed to be compensatory restored frameshifting to WT levels (Figure 6C). Similar results were also obtained for accumulation of the mutant gRNA in protoplasts (Figure 6D). These results confirm the importance of the long-distance base-pairing interaction between the RSE LB and the Pr loop for both synthesis of p94 *in vitro* and for gRNA accumulation *in vivo*.

MS residue G995 is located at the edge of the predicted pairing between the RSE and the Pr loop. As shown in Figure 3, mutating this guanylate to a cytidylate (MSm3B) reduced frameshifting by 6-fold. To determine if this reduction in p94 synthesis was due to a requirement for G995 in the long-distance interaction with the Pr loop, the G995 partner residue in the Pr loop (C4242) was changed to a guanylate to restore presumptive base-pairing. Altering the residues in this putative pair in either the RSE or Pr loop reduced frameshifting *in vitro* by 6 and 8-fold, respectively (Figure 6F) and gRNA accumulation to 5% and 8% *in vivo* (Figure 6G). Combining both alterations to re-establish pairing restored frameshifting to 84% of WT *in vitro* and accumulation to 91% of WT *in vivo*. These results strongly suggest that the original G995C alteration in MSm3B reduced frameshifting due to a disruption of the long-distance interaction with the Pr loop and not from disrupting the structure of the RSE.

### Role of the long-distance base-pairing interaction in frameshifting

**Figure 7.**
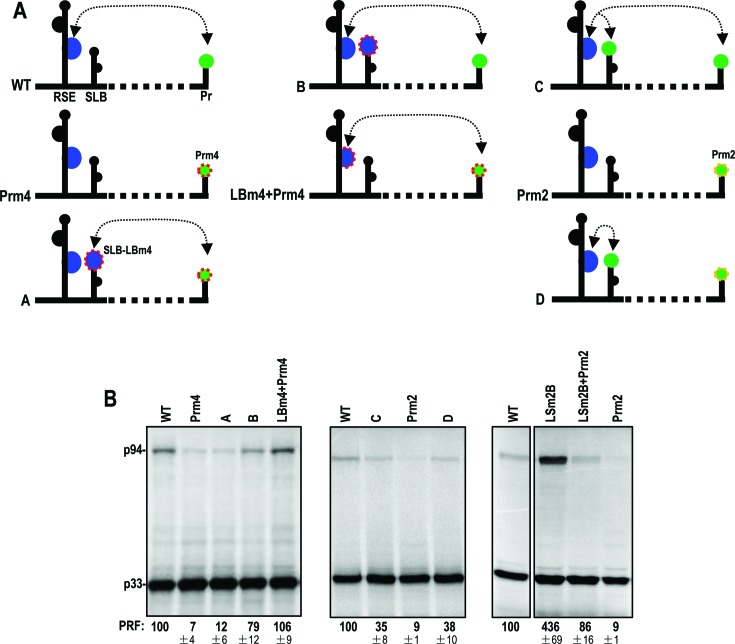
Effect of long- and short-distance interactions on frameshifting. (**A**) Schematic diagram showing alterations and putative interactions in mutant constructs. RSE LB and Pr loop sequences are colored blue and green, respectively. Known or putative base-pairing interactions are indicated by dashed arrows. (**B**) *In vitro* translation of WT and mutant gRNAs (see Figure 3 for location of LSm2B, which closes the small LS bulge and Supplementary Figures S3 and 6B for locations of Prm2 and Prm4, respectively, which disrupt the Pr loop). For image at right, all lanes are from the same phosphorimage with an irrelevant lane removed.

With the exception of GRV, all RSE long-distance interactions in Umbraviruses involve sequences at or near the 3′ terminus (Supplementary Figure S2). This suggests two possible explanations for how this interaction stimulates ribosome recoding, which are not mutually exclusive: 1) long-distance pairing of the LB with 3′ sequences is needed to alter RSE structure to support the recoding event; and/or 2) the long-distance interaction is needed to bring the 3′ terminus of the genome into proximity with the RSE. To investigate the latter possibility, the normal long-distance interaction between the RSE and the Pr loop was disrupted by 1) mutating the Pr loop (Prm4; see Figure 6B); and 2) replacing the terminal loop of the SLB hairpin just downstream of the RSE with a sequence that is perfectly complementary to the mutated Pr loop. Together, these two alterations should generate a new long-distance interaction between SLB and the Pr (Figure 7A, construct A). When compared with frameshifting by the control construct Prm4 (7% of WT), creating conditions that should bring the 3′ terminus to the vicinity of the RSE (construct A) did not significantly affect frameshifting (Figure 7B). When only the terminal loop of SLB was altered, frameshifting decreased by 21% (construct B), suggesting that there may be a small effect of this hairpin on recoding that was not evident when the entire SLB was deleted (Figure 2F).

To determine if a pseudoknot created by pairing external sequence with the RSE LB can stimulate frameshifting without concomitant relocation of the 3′ end, the terminal loop of SLB was replaced with the identical Pr loop sequence in WT gRNA (Figure 7A, construct C). This introduces a new ‘short-distance’ interaction between the RSE and SLB while simultaneously permitting the normal long-distance interaction. These mutations in construct C reduced frameshifting to 35% of WT (Figure 7B, middle), suggesting that competition for pairing with the LB was occurring and that this latter pairing was less effective than the natural pairing. When the Pr loop mutation Prm2 was added (Figure 7A, construct D), only the short distance interaction should be possible. Construct D generated 38% of WT frameshifting, a similar level as found when the long-distance interaction was also available. This supports the proposition that the new short-distance interaction between the RSE and SLB in construct C was outcompeting the long-distance interaction (Figure 7B, middle). Compared with Prm2 (no pairing with the RSE), pairing between the SLB altered loop and the RSE improved -1PRF by more than 4-fold (from 9% to 38% of WT).

To further support the proposition that the newly introduced short-distance interaction between the RSE and mutant SLB was occurring and was responsible for -1PRF enhancement in construct D, a second set of mutations were generated that disrupted the WT interaction with the Pr and created SLB as a pairing partner (Supplementary Figure S4, construct G). In this construct, the Pr contained mutation Prm2 (see Supplementary Figure S3B), the RSE contained mutation MSm3B (see Figure 6E) and the SLB terminal loop was modified to contain Pr loop sequence with the corresponding Prm3 mutation. -1PRF was enhanced by 3- to 4-fold in this construct, compared with no pairing (Supplementary Figure S4B). These results suggest that creating a short-distance pseudoknot with the RSE enhances frameshifting, but the interaction is not as stimulatory as the WT Pr interaction. The reason why the interaction with the Pr leads to more efficient frameshifting remains unknown.

One possibility for how base-pairing between the LB and a downstream sequence contributes to RSE function is that this results in stabilization of the LS. As shown in Figure 3, -1PRF efficiency was substantially enhanced by closing the small symmetrical loop and stabilizing the RSE LS. Thus, if the long-distance interaction stabilizes the LS, then an RSE modified to contain a stabilized LS might be less dependent on the long-distance interaction for function. To test this hypothesis, Pr mutation Prm2 (from Supplementary Figure S3B), which disrupts the long-distance interaction, was combined with mutation LSm2B, which closes the small symmetrical loop in the LS and enhances frameshifting by over 4-fold (Figures 3 and 7B, right). The frameshift efficiency of LSm2B was reduced by 5-fold when combined with Prm2. In contrast, WT gRNA frameshifting efficiency was reduced by 11-fold with Prm2 alone. These results indicate that an RSE with a stabilized LS still benefits from the long-distance base-pairing interaction. However, the results also suggest that the benefit of the long-distance interaction is reduced by over 2-fold when the LS is more stable.

### Importance of spacer size and p33 stop codon on -1PRF

**Figure 8.**
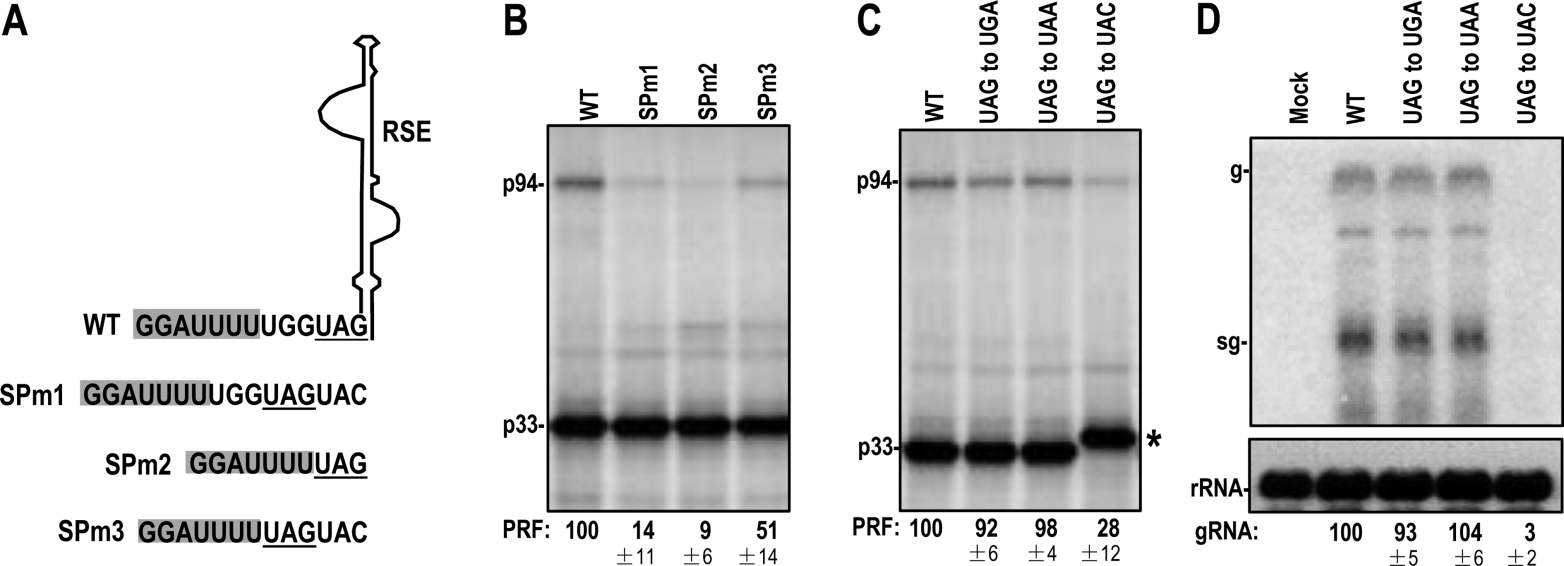
Role of spacer length and stop codon on -1PRF. (**A**) Alterations changed the distance between the slippery sequence (shaded) and RSE. p33 UAG stop codon is underlined. (**B**) and (**C**) *In vitro* translation of WT and mutant gRNAs. UAG to UAC extended the p33 ORF by 72 nt, generating a larger protein denoted by asterisk. (**D**) RNA gel blot analysis of WT and p33 stop codon mutant gRNAs in Arabidopsis protoplasts.

When a translating ribosome encounters an RSE, the ribosome must pause precisely at the slippery site where the frameshift occurs. The length of the spacer between the slippery site and the RSE, and composition of spacer nucleotides is important for optimal frameshifting in IBV, HIV and HTLV-2 (24–26). A 5-nt spacer is found for RCNMV and BYDV as well as six Umbraviruses including PEMV (Supplementary Figure S1). For PEMV, increasing or decreasing the spacer length (SPm1, 9 nt and SPm2, 2 nt, respectively) reduced -1PRF by 7- and 11-fold (Figure 8A and B). Since SPm1 both increases spacer length and moves the stop codon 3 nt from the base of the RSE, a third construct was designed that maintains the extra distance between the stop codon and the RSE but reduces the spacer length to 6 nt (SPm3). -1PRF improved by nearly 4-fold for SPm3, however frameshifting was still 2-fold less than WT. These results suggest that both maintaining an optimal spacer length and location of the stop codon at the base of the RSE is important for efficient frameshifting.

In Umbraviruses, Dianthoviruses and Luteoviruses that use -1PRF for expression of the RdRp, the stop codon of the pre-shift ORF is located either immediately downstream of the slippery site (e.g. GRV, BYDV, RCNMV) or 3 nt downstream of the slippery site (e.g. PEMV), which in all cases are adjacent to the RSE. It has been proposed that a stop codon in this location enhances ribosome slippage by introducing a pause due to entry and activity of release factors (50). For BYDV, changing the UAG stop codon to a UCG sense codon reduced -1PRF to background levels using a GUS reporter construct (5), but not in full-length viral gRNA (51). To determine if the identity or presence of the p33 stop codon in PEMV influences frameshifting efficiency in full-length gRNA, the WT UAG stop codon was replaced with alternative stop codons UGA and UAA, as well as a UAC sense codon. Note that for all three of these alterations, the lowest G:C pair in the RSE LS is disrupted. In addition, replacing the UAG with a UAC sense codon extends the p33 ORF by 72-nt. Changing the WT UAG to UGA or UAA stop codons had no discernible impact on -1PRF *in vitro* or viral gRNA accumulation *in vivo* (Figure 8C and D). Changing the stop codon to a sense codon generated an extended product when ribosomes terminated at the new location (Figure 8C, asterisk) and reduced -1PRF by more than 3-fold *in vitro*. The reduction in RdRp synthesis and/or extension of p33 reduced viral gRNA accumulation *in vivo* to background levels. These results support a positive contribution of a stop codon for frameshifting efficiency at least when located 3 nt after the slippery sequence.

### SLB enhances frameshifting in the absence of the RSE

**Figure 9.**
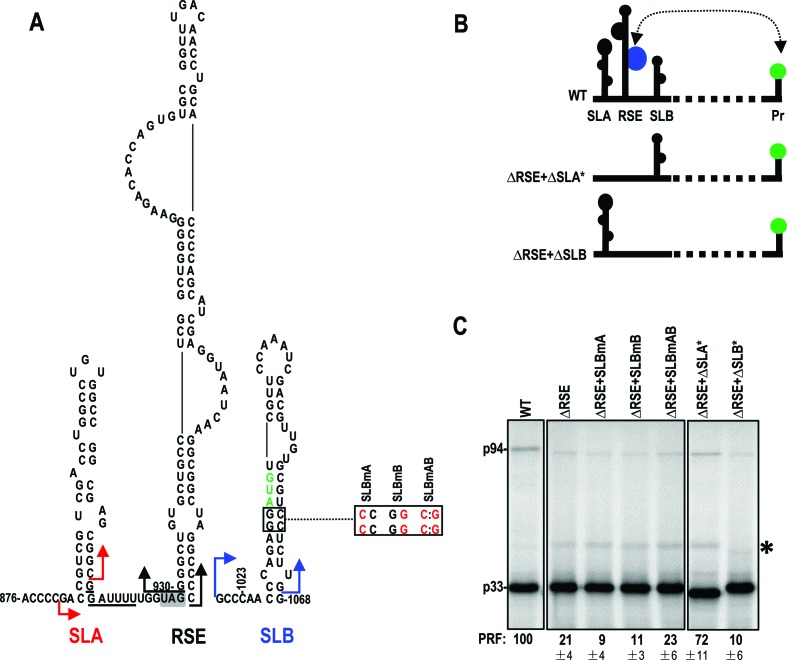
The role of SLA and SLB on -1PRF in the absence of the RSE. (**A**) Location of deletion end points for ΔSLA* (red), ΔRSE (black) and ΔSLB* (blue) used for this experiment are shown (the asterisk denotes that these deletions differ slightly from previous deletions used in Figure 2 to make the constructs more precise). Base alterations in the stem of SLB are shown. Putative AUG start codon in the -1 frame in SLB is in green. The slippery sequence is underlined. p33 UAG stop codon is shaded. (**B**) Schematic diagram showing structural elements in constructs ΔRSE+ΔSLA* and ΔRSE+ΔSLB*. LB is in blue and Pr loop is in green. (**C**) *In vitro* translation of WT and mutant gRNAs. Asterisk denotes a ≈57 kDa polypeptide that is more prominent in the absence of the RSE.

To determine if a random hairpin downstream from the slippery sequence can promote -1PRF, the RSE was deleted while maintaining the -1 frame and placing SLB just downstream from the slippery site (ΔRSE; Figure 9). Deleting the RSE, which additionally eliminates the long-distance interaction, resulted in 21% of WT levels of p94. This frameshifting amount is over 2-fold greater than the levels obtained if the LS of the RSE is disrupted (LSm1A or LSm1B, 11% and 13%, respectively; Figure 2E) or if the long-distance base-pair interaction with the Pr loop is disrupted (e.g. Prm3 or Prm4, 12% and 7%, respectively; Figure 6). To determine if SLB was contributing to this level of frameshifting in the absence of the RSE (although located 12-nt downstream from the slippery site), single and compensatory mutations were engineered into the SLB stem (SLBmA, SLBmB and SLBmAB) in the ΔRSE background (Figure 9A). Disrupting the SLB stem reduced frameshifting to 9% and 11% of WT levels, whereas reforming the stem enhanced frameshifting to 23% of WT (Figure 9C). Addition of the Prm4 interacting sequence to the loop of SLB to allow for a long-distance interaction with the 3′ end containing the Prm4 alterations in the Pr loop (Supplementary Figure S5A, construct J) did not enhance frameshifting (Supplementary Figure S5B). These results suggest that in the absence of the RSE, SLB can mediate a low level of frameshifting in the absence of a long-distance interaction with 3′ end sequence.

In the presence of the RSE, deletion of upstream hairpin SLA had no discernible effect on -1PRF in WGE (Figure 2F). Since hairpins in similar locations are conserved throughout the *Tombusviridae*, it seemed reasonable to consider that SLA might assume an as yet undetermined role in recoding. To determine if SLA might have a role in controlling frameshifting at the downstream slippery site in the absence of the RSE (e.g. when the RSE might adopt an alternative conformation), SLA was deleted in the ΔRSE background. As shown in Figure 9C, deleting both SLA and the RSE unexpectedly enhanced frameshifting to 72% of WT levels. This result suggests that in the absence of an active RSE structure, an upstream hairpin might be needed to inhibit frameshifting from a downstream slippery site.

Deletion of the RSE also caused enhanced translation of an unexpected product of about 57 kDa (p57; Figure 9C, asterisk), but not when SLB was also deleted. SLB contains an in-frame AUG codon on the 5′ side of its stem (Supplementary Figure S6A), and translation from this AUG should result in a protein of 57 kDa. Mutating this putative start codon eliminated this product *in vitro* (Supplementary Figure S6B). It remains to be determined if p57 is translated *in vivo* and if so, whether it is biologically relevant.

## DISCUSSION

### A-, P- and E-site codons contribute to frameshifting

For this report, we undertook an investigation of the sequences and structures surrounding the -1PRF site in PEMV RNA2. The PEMV slippery sequence, identified as ‘5′-GGAUUUU-3′, deviates from the conventional X XXY YYZ and allows the A-site tRNA^AAA^ to form three Watson–Crick base-pairs in the -1 frame and P-site tRNA^CUA^ to form one Watson–Crick base-pair. Since the geometry of codon:anticodon base-pairing in the P-site is not sensed as strictly as that in the A-site (52), mismatches in the P-site are tolerated (53). Mutational analysis of the PEMV slippery sequence revealed that A-site mismatches were more detrimental to frameshifting efficiency than P-site mismatches (Figure 1C). This is consistent with a recent report that used single molecule force spectroscopy to characterize single ribosome translocation dynamics when frameshifting during translation of *E.coli* dnaX mRNA (54). The authors determined that A-site mismatches tended to result in a higher percentage of premature termination events than P-site mismatches, resulting in truncated polypeptides.

Although the cognate tRNA in the A-site was most important for frameshifting in PEMV, mutations in the P-site codon reduced -1PRF by ≈60% (Figure 1C; GAU-m3 and GAU-m4). These results support a simultaneous slippage model where both P-site and A-site tRNAs contribute to recoding (10,21). In contrast, the P-site slippage model proposed for *Potato virus M* (PMV; family *Betaflexiviridae*) only required a 4-nt sequence (AAAA) when the peptidyl-tRNA slips in the P-site (55). E-site mutation GCG-m1 also reduced frameshifting by 60% (Figure 1C), despite not affecting E-site tRNA re-pairing. This result is in agreement with a previous report for HIV, where mutating the E-site codon also alters -1PRF efficiency (49). For HIV, the impact of alterations in the E-site codon on -1PRF persisted even when the slippery sequence was replaced with that of *Giardia virus, Equine infectious anemia virus* and SARS-CoV (49). Since there was no consistent correlation between frameshift efficiency and mismatches in the E-site, the authors proposed that structural peculiarities of the E-site tRNA, other than codon-anticodon base-pairing, affected -1PRF by interfering with movement of the A- and P-site tRNAs (49).

### The PEMV RSE has a complex higher order structure

Phylogenetic analysis combined with SHAPE structure probing identified a hairpin with two asymmetric bulges immediately downstream from the p33 termination codon that strongly stimulates frameshifting. The PEMV RSE resembles the -1PRF recoding elements required for RdRp synthesis in BYDV and RCNMV, as well as the hairpin necessary for translational readthrough in TNV-D, CIRV and TCV (6,33–35). Despite the general conservation of RSE structures throughout the *Tombusviridae*, and the LSm1 series of single and compensatory mutations that confirmed the importance of the RSE LS for frameshifting, SHAPE profiles for mutants with altered LS (LSm1) did not support the phylogenetically conserved RSE structure. Instead, SHAPE results strongly suggested that the gRNA synthesized *in vitro* adopts a complex higher order structure that connects both sides of the LS base with the lower region of the MS and the spacer sequence upstream of the RSE (Figure 4A and B). Altering the upper MS also affected several residues in the UB, suggesting that higher order structure may exist throughout the RSE that forms in *in vitro* synthesized transcripts (Figure 5A and B).

There are several possible explanations for the conflict between the genetic analyses/phylogenetic structure and the SHAPE profiles: (i) *In vitro* SHAPE and WGE reactions contain different factors and ion concentrations, which might impact RNA folding and result in different RNA conformations in the two assays; (ii) the elongating ribosome on the gRNA template might affect the folding/refolding of structures; or (iii) the PEMV RSE may functionally exist in more than one conformation—e.g. an inactive ‘basal’ conformation and an active phylogenetically—conserved structure, as was recently found for HIV (23). An additional possibility is that the different temperatures used for WGE (25°C) and SHAPE (37°C) contributed to the difference. However, this is unlikely as SHAPE of the RSE region conducted at 25°C was essentially identical (data not shown). A similar conflict between genetic analyses and SHAPE data was also recently found for the TCV RSE, which has a critical alternative (basal) conformation that also forms *in vivo* (M. Khulmann and AE Simon, manuscript in preparation). Because of the complexity of the SHAPE profiles in the current study, we were unable to determine the structure of this putative alternative conformation for the PEMV RSE.

RSE have been proposed to function as roadblocks that cause a translating ribosome to pause at the slippery site, with the basal stem of the RSE contacting the ribosome at the entrance to the mRNA tunnel (56,57). For translation to continue, ribosomes must overcome the resistance imposed by a base-paired or pseudoknotted RSE structure by unwinding the paired bases (58,59). Mechanically stable LS should provide greater resistance to ribosome unwinding, thus creating conditions whereby a subset of ribosomes undergo incomplete translocation, resulting in a frameshift. For HIV, a correlation was found between frameshifting efficiency and stability of the first 3–4 bp of the lower stem but not the overall thermodynamic stability of the RSE (60). The same trend of frameshifting efficiency versus stability of the stimulatory element was reported for antisense oligonucleotides and RNA G-quadruplex structures (30,61). Strengthening the PEMV LS stem by closing the small symmetrical loop (LSm2A and B) enhanced frameshifting by 2- and 4-fold, respectively. However, LSm2AB, with G A and U C in the loop also gave enhanced frameshifting. According to SHAPE, the flexibility of these loop residues in LSm2AB was similar to WT (Figure 4D), suggesting that at least for this conformation, stem stability was likely similar to WT. It may be that, similar to when RNA pseudoknotted structures are used as an RSE for -1PRF, conformational plasticity of the RNA structure, as opposed to strict mechanical stability, is an important determinant for -1PRF efficiency (62).

### Long-distance interaction with the RSE

PEMV RSE activity is dependent on a long-distance base-pairing interaction with a sequence near the 3′ terminus of the gRNA, and this interaction (involving different primary sequences) is conserved in other Umbraviruses and many other plant viruses (Supplementary Figure S2) (6,33–35). The mechanism by which this long-distance interaction contributes to -1PRF is not known. We investigated two possibilities: a requirement to bring the 3′ terminus to the vicinity of the RSE; and as a means of modifying the structure of the RSE to promote the frameshift event. Disrupting the interaction with the RSE and allowing for a similar interaction with a nearby downstream hairpin (SLB) did not significantly stimulate -1PRF (Figure 7B, construct A). However, replacing the long-distance base-pairing interaction between the RSE and Pr with a short-distance interaction between the RSE and SLB improved -1 PRF by more than 4-fold over levels obtained in the absence of any long-distance interaction (Figure 7B middle, construct D). In addition, RSE with a base-paired lower symmetrical bulge was more tolerant to the loss of the long-distance interaction (Figure 7B, right). These results suggest that the long-distance interaction modifies the structure of the RSE, possibly by strengthening the stability of the lower stem. However, we cannot eliminate the possibility that spatial proximity of the 3′ end also contributes to frameshifting as there is no direct evidence that the artificial interaction between SLB and Pr occurs. We have also been unsuccessful at visualizing the WT RSE-Pr interaction by SHAPE (data not shown), suggesting either that formation of the long-distance interaction naturally requires additional ribosome-mediated events/host factors or that only a small percent of gRNA in the population contain the interaction.

### Contribution of a stop codon to frameshifting

Although amber is the universal stop codon at the base of RSE in the *Tombusviridae*, replacement with the other two stop codons in PEMV gRNA had no discernable effect *in vitro* or *in vivo* (Figure 8C and D). In contract, replacement of the stop codon with a sense codon reduced frameshifting by 72% *in vitro*. How a stop codon at the base of the RSE contributes to frameshifting is not known. The lack of a third position guanylate to close the RSE LS was likely not a factor, since gRNA with UGA and UAA stop codons were also missing the terminal LS base-pair. In addition, since the ribosome would be past the slippery site when the stop codon enters the A-site, an additional pause due to entry of release factors would not benefit frameshifting directly. Alternatively, it is possible that the stop codon affects the density of polysomes near the slippery site by causing ribosome stacking behind the ribosome in the process of terminating translation. When the terminating ribosome is released, the next ribosome accommodates the slippery sequence and encounters the RSE while translating at a slower pace than ribosomes not in the vicinity of a stop codon. The RSE may then restrict slow moving ribosomes more efficiently, thus positively impacting frameshifting. Similar to PEMV, the UGA stop codon following frameshifting in PVM (AAAAUGA) could be replaced with the other two stop codons but not a sense codon when assayed in a reporter construct (55). In contrast, alteration of the far downstream *gag* protein UAG stop codon to a sense codon in *Rous sarcoma virus* did not impact frameshifting (10).

### Possible role for the phylogenetically conserved SLA hairpin in frameshifting

Deletion of the PEMV RSE decreased frameshifting by ≈5-fold (Figure 9C), similar to what was found for HIV and HTLV-2 (25,63). A portion of the residual frameshifting activity in PEMV was attributed to SLB that was newly located 12-nt downstream of the slippery site (Figure 9C). The weak stimulation by SLB was not due to the longer than optimal spacer length as reducing the spacer length to 6 nt did not improve frameshifting efficiency (data not shown).

The presence of a hairpin just upstream of the RSE (SLA) is ubiquitous throughout the *Tombusviridae*, regardless of whether the virus uses frameshifting or readthrough to produce the RdRp. Such strict conservation of a cis-acting element suggests an important function of the hairpin related to recoding. Deletion of SLA had no obvious effect on frameshifting efficiency in WGE. However, our finding of residual frameshifting activity in the absence of the RSE and the likelihood that an alternative structure exists for portions of the RSE led to the possibility that SLA might be needed to suppress RdRp synthesis under conditions when the phylogenetically conserved RSE conformation was not present and RdRp synthesis might be detrimental. Deletion of SLA enhanced frameshifting to 72% of WT in the ΔRSE background (Figure 9C; ΔRSE versus ΔRSE+ΔSLA*), strongly suggesting that a hairpin upstream of the slippery site suppresses downstream frameshifting when an RSE is not present (or not formed). A previous study also revealed a suppressive role for a hairpin located 4-nt upstream of the slippery site in SARS-CoV, and this hairpin was able to suppress frameshifting stimulated by pseudoknots from a number of different viruses (31,64). The level of attenuation reported was sensitive to the stability of the stem as well as the distance from the slippery site (64). In PEMV, the basal portion of the SLA stem would be melted when a ribosome is positioned at the slippery sequence, but the remainder of the hairpin may provide mechanical resistance for 5′ movement of the ribosome, thus suppressing frameshifting. However, such a suppression mechanism must not occur when the RSE is present. One possibility is that the PEMV RSE when engaged in the long-distance interaction with the 3′ Pr is so effective at stimulating frameshifting that resistance from SLA is negligible. Since most studies on RSE involve excision of the RSE from its *in situ* location and insertion within artificial reporter constructs for subsequent analyses, similar suppressive hairpins (and long-distance interactions) associated with ribosome recoding may have been overlooked.

## Supplementary Material

SUPPLEMENTARY DATA
