# Supplementary material for: Multiple Cis-acting elements modulate programmed -1 ribosomal frameshifting in Pea enation mosaic virus
Source: Nucleic Acids Res. 2015 Nov 17;44(2):878–95. doi: 10.1093/nar/gkv1241 (PMC4737148; doi:10.1093/nar/gkv1241)
Supplement: SUPPLEMENTARY DATA [file supp_44_2_878__index.html]

Multiple Cis-acting elements modulate programmed -1 ribosomal frameshifting in Pea enation mosaic virus — SUPPLEMENTARY DATA 

# Multiple Cis-acting elements modulate programmed -1 ribosomal frameshifting in Pea enation mosaic virus

## SUPPLEMENTARY DATA

- SUPPLEMENTARY DATA
