# Supplementary material for: Multiple Cis-acting elements modulate programmed -1 ribosomal frameshifting in Pea enation mosaic virus
Source: Nucleic Acids Res. 2015 Nov 17;44(2):878–95. doi: 10.1093/nar/gkv1241 (PMC4737148; doi:10.1093/nar/gkv1241)
Supplement: SUPPLEMENTARY DATA [file supp_gkv1241_nar-02985-a-2015-File003.pptx]

## Slide 1
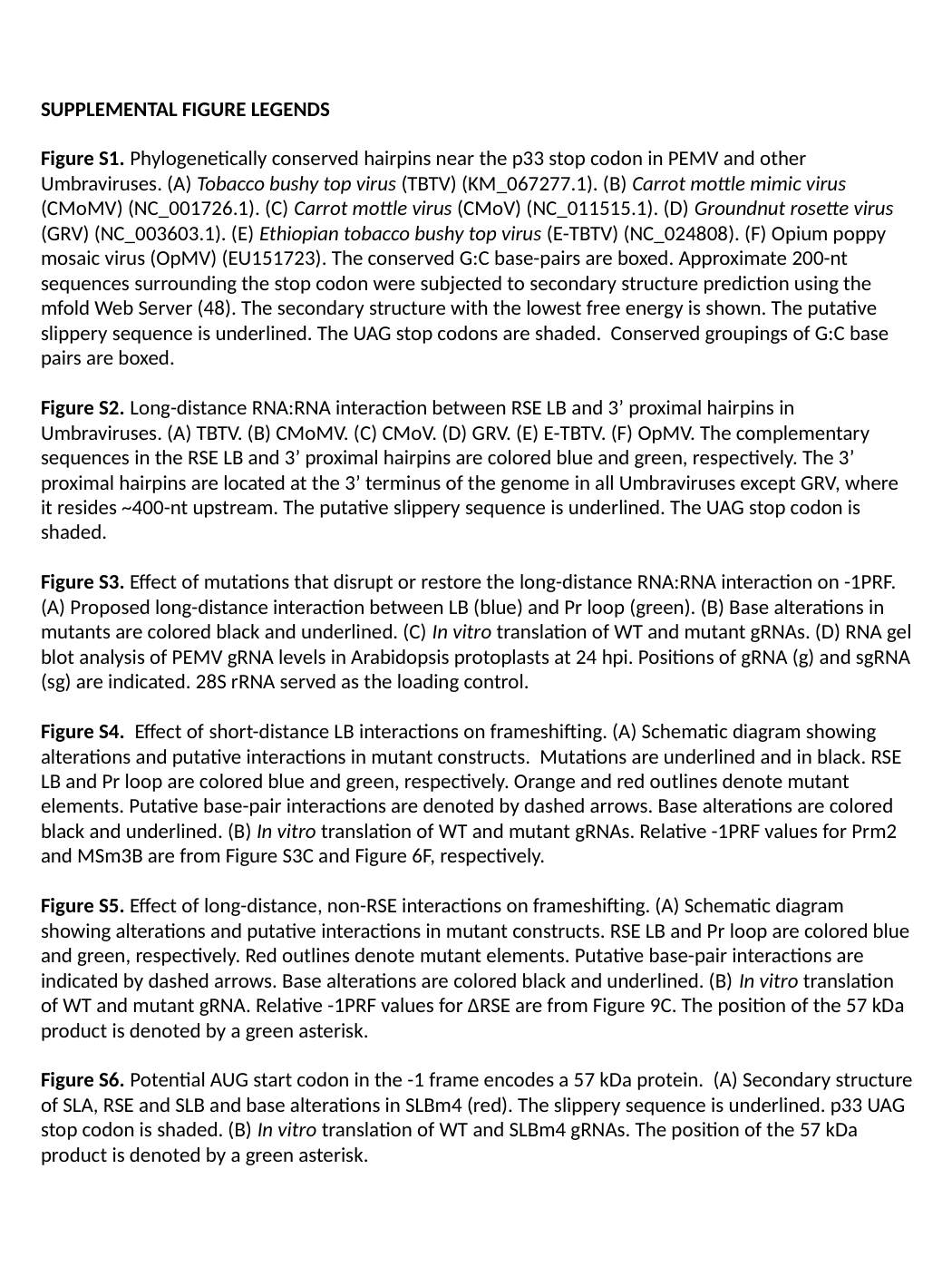

Supplemental Figure Legends
Figure S1. Phylogenetically conserved hairpins near the p33 stop codon in PEMV and other Umbraviruses. (A) Tobacco bushy top virus (TBTV) (KM_067277.1). (B) Carrot mottle mimic virus (CMoMV) (NC_001726.1). (C) Carrot mottle virus (CMoV) (NC_011515.1). (D) Groundnut rosette virus (GRV) (NC_003603.1). (E) Ethiopian tobacco bushy top virus (E-TBTV) (NC_024808). (F) Opium poppy mosaic virus (OpMV) (EU151723). The conserved G:C base-pairs are boxed. Approximate 200-nt sequences surrounding the stop codon were subjected to secondary structure prediction using the mfold Web Server (48). The secondary structure with the lowest free energy is shown. The putative slippery sequence is underlined. The UAG stop codons are shaded. Conserved groupings of G:C base pairs are boxed.
Figure S2. Long-distance RNA:RNA interaction between RSE LB and 3’ proximal hairpins in Umbraviruses. (A) TBTV. (B) CMoMV. (C) CMoV. (D) GRV. (E) E-TBTV. (F) OpMV. The complementary sequences in the RSE LB and 3’ proximal hairpins are colored blue and green, respectively. The 3’ proximal hairpins are located at the 3’ terminus of the genome in all Umbraviruses except GRV, where it resides ~400-nt upstream. The putative slippery sequence is underlined. The UAG stop codon is shaded.
Figure S3. Effect of mutations that disrupt or restore the long-distance RNA:RNA interaction on -1PRF. (A) Proposed long-distance interaction between LB (blue) and Pr loop (green). (B) Base alterations in mutants are colored black and underlined. (C) In vitro translation of WT and mutant gRNAs. (D) RNA gel blot analysis of PEMV gRNA levels in Arabidopsis protoplasts at 24 hpi. Positions of gRNA (g) and sgRNA (sg) are indicated. 28S rRNA served as the loading control.
Figure S4. Effect of short-distance LB interactions on frameshifting. (A) Schematic diagram showing alterations and putative interactions in mutant constructs. Mutations are underlined and in black. RSE LB and Pr loop are colored blue and green, respectively. Orange and red outlines denote mutant elements. Putative base-pair interactions are denoted by dashed arrows. Base alterations are colored black and underlined. (B) In vitro translation of WT and mutant gRNAs. Relative -1PRF values for Prm2 and MSm3B are from Figure S3C and Figure 6F, respectively.
Figure S5. Effect of long-distance, non-RSE interactions on frameshifting. (A) Schematic diagram showing alterations and putative interactions in mutant constructs. RSE LB and Pr loop are colored blue and green, respectively. Red outlines denote mutant elements. Putative base-pair interactions are indicated by dashed arrows. Base alterations are colored black and underlined. (B) In vitro translation of WT and mutant gRNA. Relative -1PRF values for ∆RSE are from Figure 9C. The position of the 57 kDa product is denoted by a green asterisk.
Figure S6. Potential AUG start codon in the -1 frame encodes a 57 kDa protein. (A) Secondary structure of SLA, RSE and SLB and base alterations in SLBm4 (red). The slippery sequence is underlined. p33 UAG stop codon is shaded. (B) In vitro translation of WT and SLBm4 gRNAs. The position of the 57 kDa product is denoted by a green asterisk.

## Slide 2
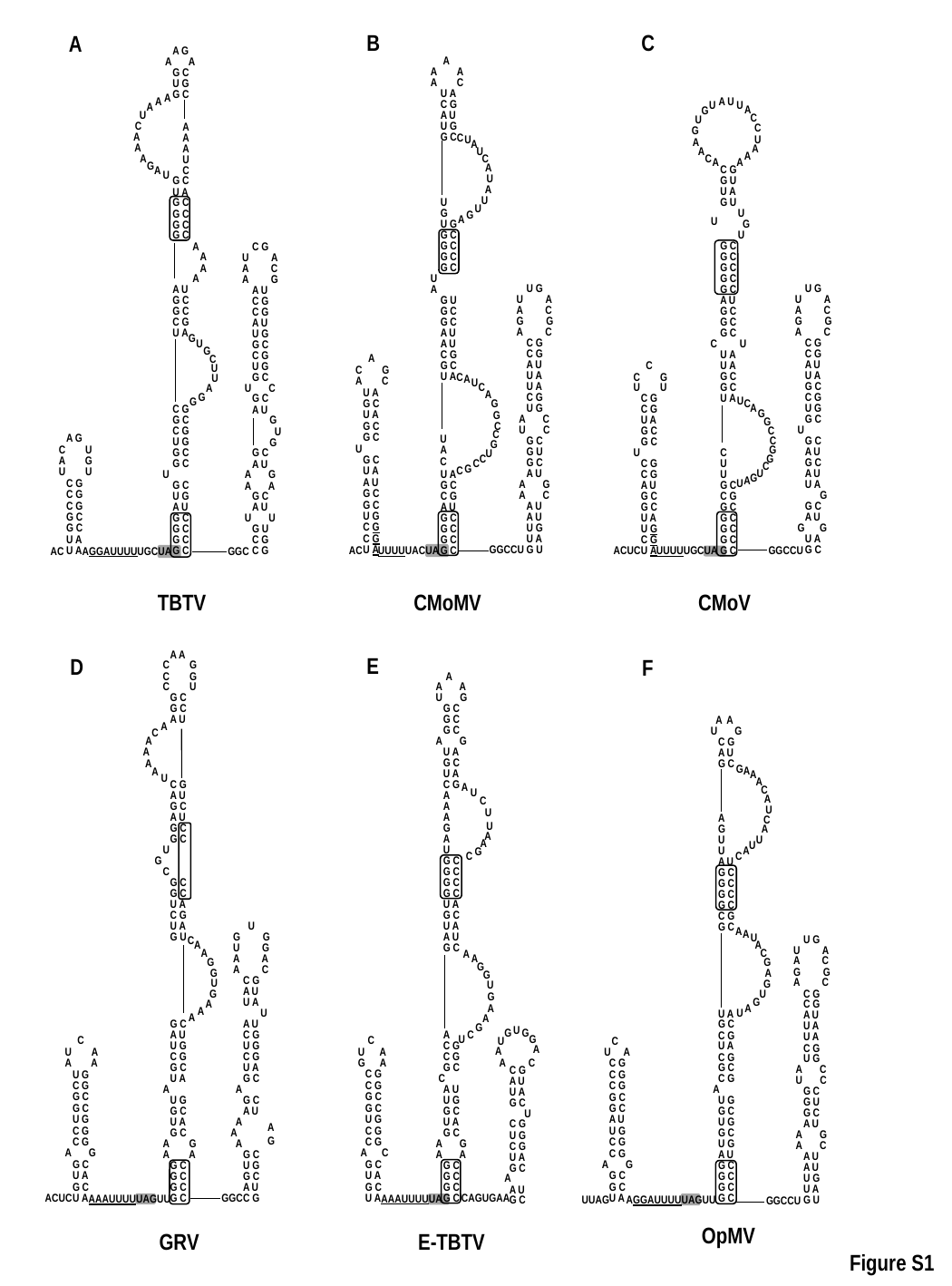

B
C
A
 A
 A A
 A C
 U A
 C G
 A U
 U G
 G C
 U
 G
 U G
 G C
 G C
 G C
 G C
 U
 A
 G U
 G C
 G C
 A U
 A U
 C G
 G C
 U A
 U A
 G C
 C G
 A U
 G C
 G C
 G C
 G C
C
U
A
U
C
A
U
A
U
U
G
A
 U G
 U A
 A C
 G G
 A C
 C G
 C G
 A U
 U A
 U A
 C G
 U G
 A C
 U C
 G C
 G U
 G C
 A U
 A G
 A C
 A U
 A U
 U G
 U A
 G U
 A
C G
A C
 U A
 G C
 U A
 G C
 G C
U
 G C
 U A
 A U
 G C
 G C
 U G
 C G
 C G
 U A
C
A
U
C
A
G
G
C
C
U
G
A
U
C
C
C
G
C
GGCCU
AC
UUUUUACUA
 A G
 A A
 G C
 U G
 G C
 A
 A
 A
 U
 C
 G C
 U A
 G C
 G C
 G C
 G C
 A
 A
 A
 A
 A U
 G C
 G C
 C G
 U A
 C G
 G C
 C G
 U G
 G C
 G C
 U
 G C
 U G
 A U
 G C
 G C
 G C
 G C
A
A
A
U
U
U
A
G
C
U
C
G
U
A
A
A
A
C
A
A
 C G
 G U
 U A
 G U
 U
 G
 U
 G C
 G C
 G C
 G C
 G C
 A U
 G C
 G C
 G C
 C U
 U A
 U A
 G C
 G C
 U A
 C
 U
 U
 G C
 C G
 G C
 G C
 G C
 G C
 G C
U
U
C
A
G
G
C
C
G
G
C
U
G
A
U
 U G
 U A
 A C
 G G
 A C
 C G
 C G
 A U
 U A
 G C
 C G
 U G
 G C
 U
 G C
 A U
 G C
 A U
 U A
 G
 G C
 A U
 G G
 U A
 G C
 C
C G
U U
 C G
 C G
 U A
 G C
 G C
U
 C G
 C G
 A U
 G C
 G C
 U A
 U G
 C G
 U A
GGCCU
ACUC
UUUUUGCUA
A
U
C
A
A
A
G
A
U
 C G
 U A
 A C
 A G
 A U
 C G
 C G
 A U
 U G
 G C
 C G
 U G
 G C
 U C
 G C
 A U
 G
 U
 G
 G C
 A U
 A G
 A A
 G C
 A U
 U U
 G U
 C G
 C G
GGC
G
U
G
C
U
U
A
G
G
 A G
C U
A G
U U
 C G
 C G
 C G
 G C
 G C
 U A
 U A
AC
AGGAUUUUUGCUA
 CMoV
 TBTV
 CMoMV
 A A
C G
C G
C U
 G C
 G C
 A U
 C G
 A U
 G C
 A U
 G C
 G C
U
C
 G C
 G C
 U A
 C G
 U A
 G U
 G C
 A U
 U G
 C G
 G C
 U A
A
 U G
 G C
 U A
 G C
A G
A A
 G C
 G C
 G C
 G C
A
C
A
A
A
A
U
G
C
A
A
G
G
U
G
A
A
A
 C
U A
A A
 U G
 C G
 G C
 G C
 U G
 C G
 C G
A G
 G C
 U A
 G C
 U A
A
G
ACUC
AAAUUUUUAGUU
 U
 G G
 U G
 A A
 A C
 C G
 A U
 U A
 U
 A U
 C G
 U G
 C G
 U A
 G C
 A
 G C
 A U
 A
 A
 A
 G C
 U G
 G C
 A U
 C G
GGC
E
D
F
 A
A A
U G
 G C
 G C
 G C
A G
 U A
 G C
 U A
 C G
 A
 A
 A
 G
 A
 U
 G C
 G C
 G C
 G C
 U A
 G C
 U A
 A U
 G C
 A
 C G
 C G
 G C
 C
 A U
 U G
 G C
 U A
 G C
A G
A A
 G C
 G C
 G C
 G C
A
U
C
U
U
A
A
G
C
A
A
G
G
U
G
A
A
G
U
G
G
C
 C G
 A U
 U A
 G C
 U
 C G
 U G
 C G
 U A
 G C
 A
 A U
 G C
U
G
U
 C
U A
G A
 C G
 C G
 G C
 G C
 U G
 C G
 C G
 A C
 G C
 U A
 G C
 U A
A
A
A
C
AAAUUUUUA
CAGUGAA
 A A
 U G
 C G
 A U
 G C
 A
 G
 U
 U
 A U
 G C
 G C
 G C
 G C
 C G
 G C
 U A
 G C
 C G
 U A
 C G
 G C
 C G
 A
 U G
 G C
 U G
 G C
 U G
 A U
 G C
 G C
 G C
 G C
G
A
A
A
C
A
U
C
A
U
U
A
C
 C
 U A
 C G
 C G
 C G
 G C
 G C
 A U
 U G
 C G
 C G
A G
 G C
 G C
 U A
A
A
U
 U G
 U A
 A C
 G G
 A C
 C G
 C G
 A U
 U A
 U A
 C G
 U G
 A C
 U C
 G C
 G U
 G C
 A U
 A G
 A C
 A U
 A U
 U G
 U A
 G U
A
C
G
A
G
U
G
A
U
AGGAUUUUUAGUU
GGCCU
UUAG
 OpMV
 GRV
 E-TBTV
Figure S1

## Slide 3
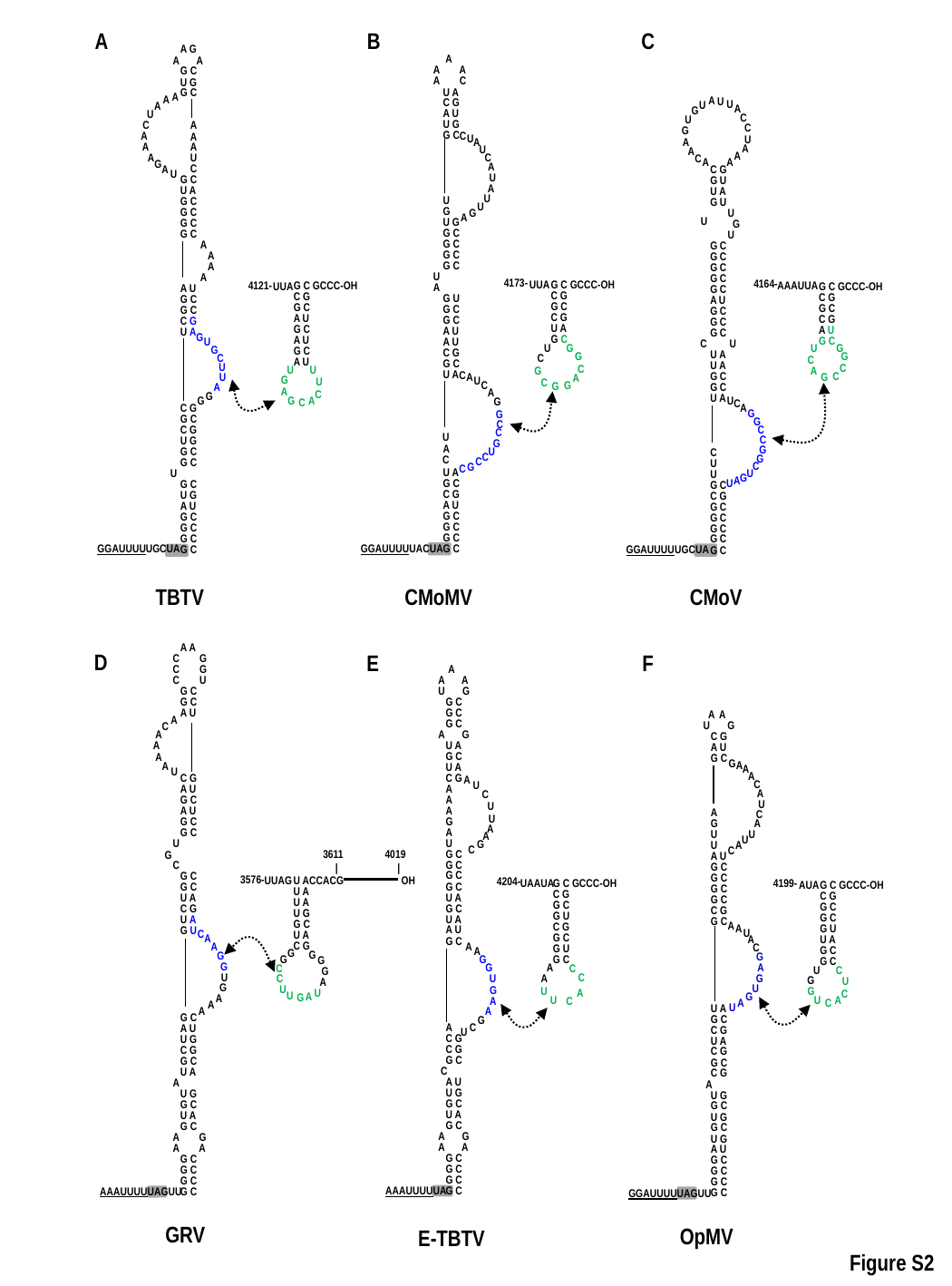

A
 A G
 A A
 G C
 U G
 G C
 A
 A
 A
 U
 C
 G C
 U A
 G C
 G C
 G C
 G C
 A
 A
 A
 A
 A U
 G C
 G C
 C G
 U A
 C G
 G C
 C G
 U G
 G C
 G C
 U
 G C
 U G
 A U
 G C
 G C
 G C
 G C
A
A
A
U
C
A
A
A
G
A
U
G
U
G
C
U
U
A
G
G
GGAUUUUUGCUA
4121-
UUA
G C GCCC-OH
C G
G C
A U
G C
A U
G C
A U
U
U
G
U
A
C
G
A
C
 TBTV
B
C
A
U
U
U
A
G
C
U
C
G
U
A
A
A
A
C
A
A
 C G
 G U
 U A
 G U
 U
 G
 U
 G C
 G C
 G C
 G C
 G C
 A U
 G C
 G C
 G C
 C U
 U A
 U A
 G C
 G C
 U A
 C
 U
 U
 G C
 C G
 G C
 G C
 G C
 G C
 G C
U
4164-
AAAUUA
G C GCCC-OH
C G
G C
C G
A U
G C
G
U
G
C
C
A
C
G
U
C
A
G
G
C
C
G
G
C
U
G
A
U
GGAUUUUUGCUA
 CMoV
 A
 A A
 A C
 U A
 C G
 A U
 U G
 G C
 U
 G
 U G
 G C
 G C
 G C
 G C
 U
 A
 G U
 G C
 G C
 A U
 A U
 C G
 G C
 U A
 U A
 G C
 C G
 A U
 G C
 G C
 G C
 G C
C
U
A
U
C
A
U
A
U
U
G
A
C
A
U
C
A
G
G
C
C
U
G
A
U
C
C
C
G
C
GGAUUUUUACUA
4173-
UUA
G C GCCC-OH
C G
G C
C G
U A
G C
G
U
G
C
C
G
A
C
G
G
 CMoMV
 A A
C G
C G
C U
 G C
 G C
 A U
 C G
 A U
 G C
 A U
 G C
 G C
U
C
 G C
 G C
 U A
 C G
 U A
 G U
 G C
 A U
 U G
 C G
 G C
 U A
A
 U G
 G C
 U A
 G C
A G
A A
 G C
 G C
 G C
 G C
A
C
A
A
A
A
U
G
C
A
A
G
G
U
G
A
A
A
AAAUUUUUAGUU
D
3611
 |
4019
 |
3576-
UUAG
U ACCACG OH
U A
U A
U G
G C
U A
C G
G
G
G
G
C
G
C
A
U
U
U
A
G
E
F
 A
A A
U G
 G C
 G C
 G C
A G
 U A
 G C
 U A
 C G
 A
 A
 A
 G
 A
 U
 G C
 G C
 G C
 G C
 U A
 G C
 U A
 A U
 G C
 A
 C G
 C G
 G C
 C
 A U
 U G
 G C
 U A
 G C
A G
A A
 G C
 G C
 G C
 G C
 A A
 U G
 C G
 A U
 G C
 A
 G
 U
 U
 A U
 G C
 G C
 G C
 G C
 C G
 G C
 U A
 G C
 C G
 U A
 C G
 G C
 C G
 A
 U G
 G C
 U G
 G C
 U G
 A U
 G C
 G C
 G C
 G C
G
A
A
A
A
C
U
A
C
U
U
C
U
A
A
U
A
U
G
A
C
C
4204-
UAAUA
G C GCCC-OH
C G
G C
G U
C G
G C
G U
G C
A
C
C
A
U
A
U
C
4199-
AUA
G C GCCC-OH
C G
G C
G C
G U
U A
G C
G C
C
U
G
U
G
C
U
A
C
A
A
U
A
A
C
A
G
G
A
G
U
G
U
G
G
A
A
U
A
G
C
U
AAAUUUUUA
GGAUUUUUAGUU
 GRV
 OpMV
 E-TBTV
Figure S2

## Slide 4
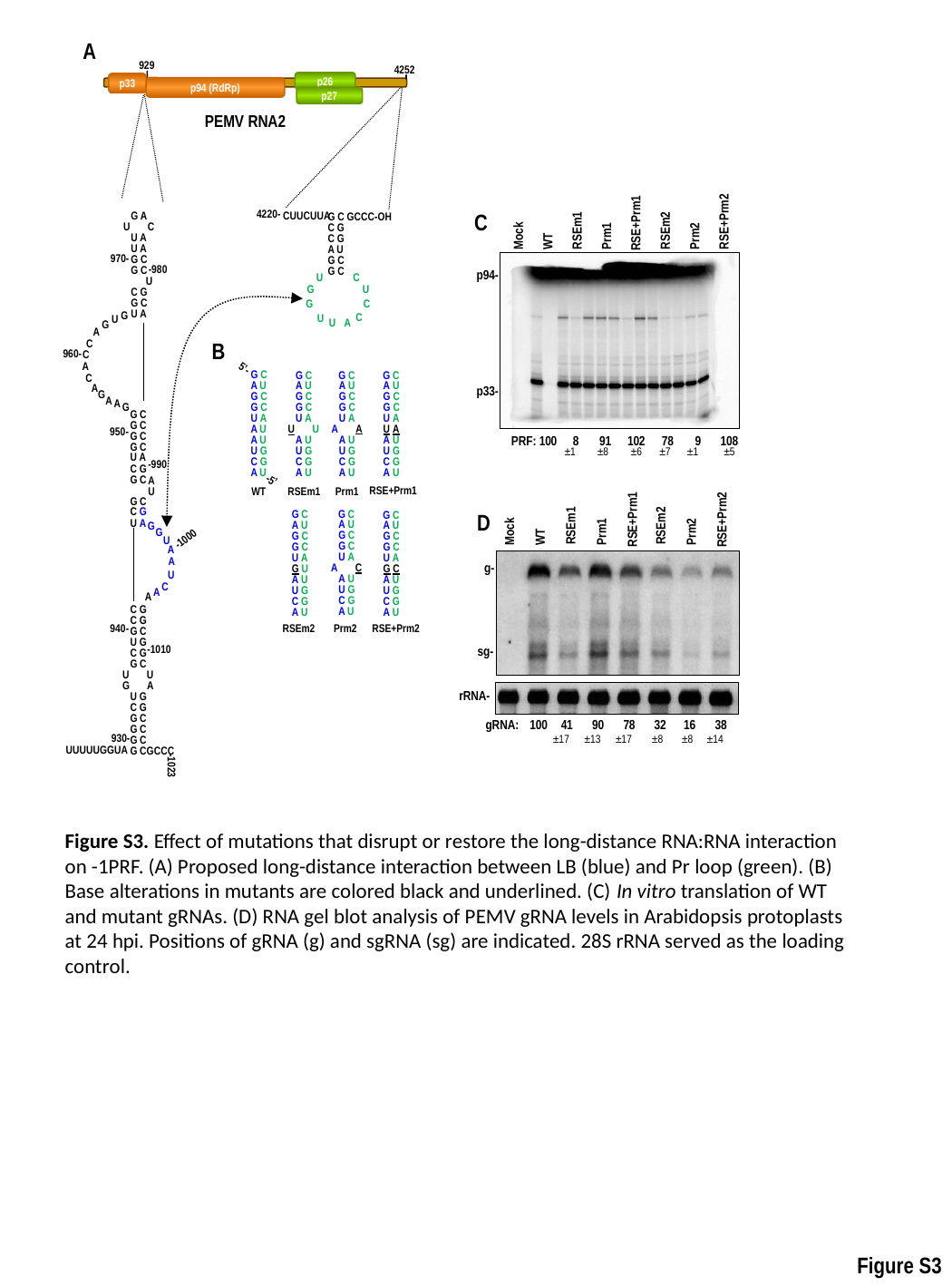

A
929
4252
p26
p33
p94 (RdRp)
p27
PEMV RNA2
4220-
C
CUUCUUA
 G A
U C
 U A
 U A
 G C
 G C
 U
 C G
 G C
 U A
 G C
 G C
 G C
 G C
 U A
 C G
 G C
 G C
 C G
 U A
 C G
 C G
 G C
 U G
 C G
 G C
 U U
 G A
 U G
 C G
 G C
 G C
 G C
 G CGCCC
G
U
G
A
C
C
A
C
A
G
A
A
G
A
U
G
G
U
A
A
U
C
A
A
UUUUUGGUA
970-
-980
960-
950-
-990
-1000
940-
-1010
930-
-1023
G C GCCC-OH
C G
C G
A U
G C
G C
RSE+Prm2
RSE+Prm1
RSEm1
RSEm2
Mock
Prm1
Prm2
WT
p94-
U
C
G
U
G
C
C
U
U
A
B
5’-
G C
 A U
 G C
 G C
 U A
 U U
 A U
 U G
 C G
 A U
RSEm1
G C
G C
 A U
 G C
 G C
 U A
 A A
 A U
 U G
 C G
 A U
Prm1
 G C
 A U
 G C
 G C
 U A
 A U
 A U
 U G
 C G
 A U
 A U
 G C
 G C
 U A
 U A
 A U
 U G
 C G
 A U
RSE+Prm1
p33-
PRF:
100
8
91
102
78
9
108
±1
±8
±6
±7
±1
±5
-5’
WT
G C
G C
 A U
 G C
 G C
 U A
 G U
 A U
 U G
 C G
 A U
RSEm2
G C
 A U
 G C
 G C
 U A
 G C
 A U
 U G
 C G
 A U
 RSE+Prm2
D
RSE+Prm1
RSE+Prm2
RSEm1
RSEm2
 A U
 G C
 G C
 U A
 A C
 A U
 U G
 C G
 A U
Prm2
Mock
Prm1
Prm2
WT
g-
sg-
rRNA-
gRNA:
100
41
90
78
32
16
38
±17
±13
±17
±8
±8
±14
Figure S3. Effect of mutations that disrupt or restore the long-distance RNA:RNA interaction on -1PRF. (A) Proposed long-distance interaction between LB (blue) and Pr loop (green). (B) Base alterations in mutants are colored black and underlined. (C) In vitro translation of WT and mutant gRNAs. (D) RNA gel blot analysis of PEMV gRNA levels in Arabidopsis protoplasts at 24 hpi. Positions of gRNA (g) and sgRNA (sg) are indicated. 28S rRNA served as the loading control.
Figure S3

## Slide 5
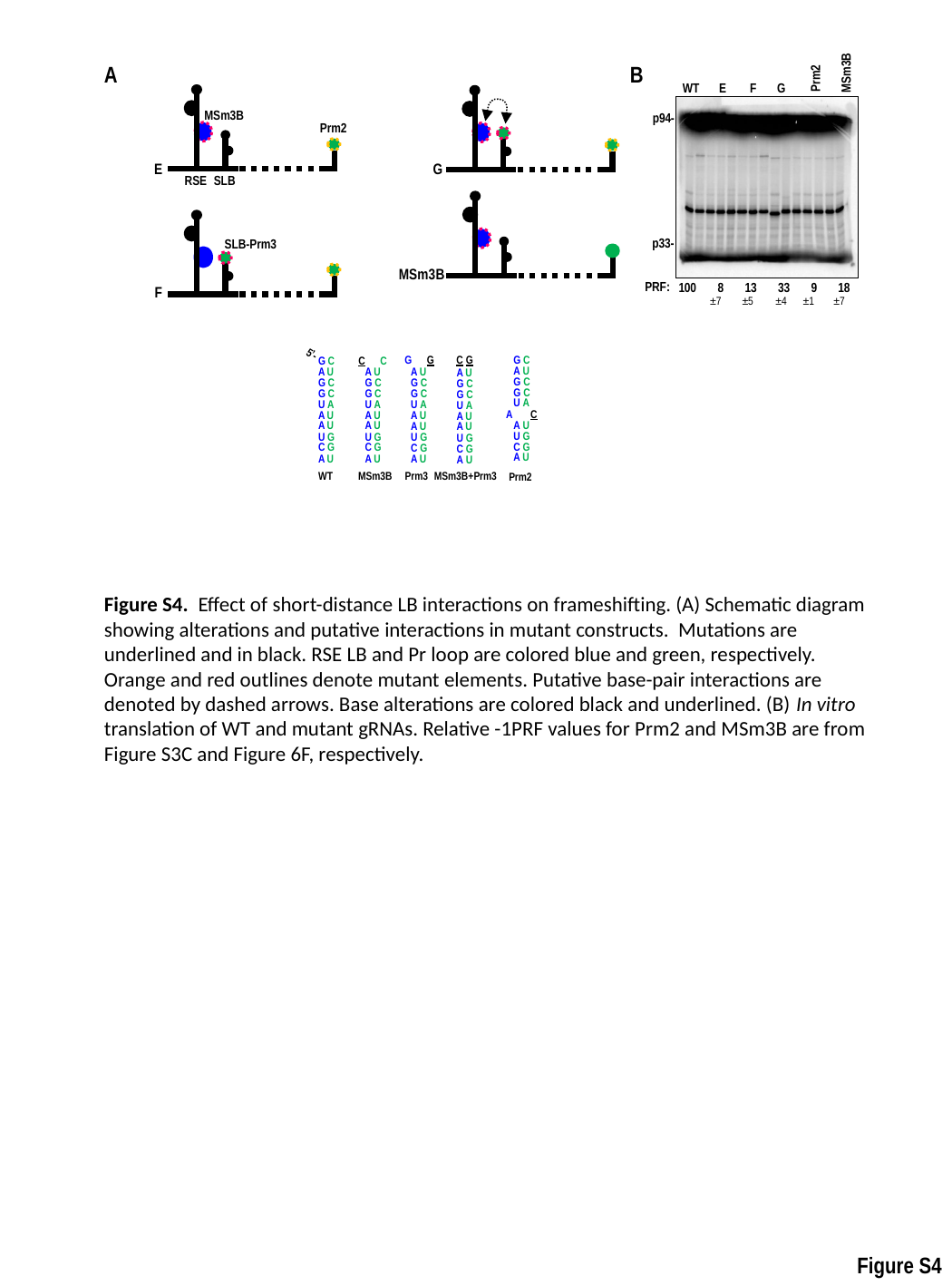

B
A
MSm3B
Prm2
WT
E
F
G
MSm3B
p94-
Prm2
E
G
RSE
SLB
p33-
SLB-Prm3
MSm3B
PRF:
100
8
13
33
9
18
F
±7
±5
±4
±1
±7
5’-
C G
G G
C C
 G C
 A U
 G C
 G C
 U A
 A C
 A U
 U G
 C G
 A U
 A U
 G C
 G C
 U A
 A U
 A U
 U G
 C G
 A U
 G C
 A U
 G C
 G C
 U A
 A U
 A U
 U G
 C G
 A U
 A U
 G C
 G C
 U A
 A U
 A U
 U G
 C G
 A U
 A U
 G C
 G C
 U A
 A U
 A U
 U G
 C G
 A U
WT
MSm3B
Prm3
MSm3B+Prm3
Prm2
Figure S4. Effect of short-distance LB interactions on frameshifting. (A) Schematic diagram showing alterations and putative interactions in mutant constructs. Mutations are underlined and in black. RSE LB and Pr loop are colored blue and green, respectively. Orange and red outlines denote mutant elements. Putative base-pair interactions are denoted by dashed arrows. Base alterations are colored black and underlined. (B) In vitro translation of WT and mutant gRNAs. Relative -1PRF values for Prm2 and MSm3B are from Figure S3C and Figure 6F, respectively.
Figure S4

## Slide 6
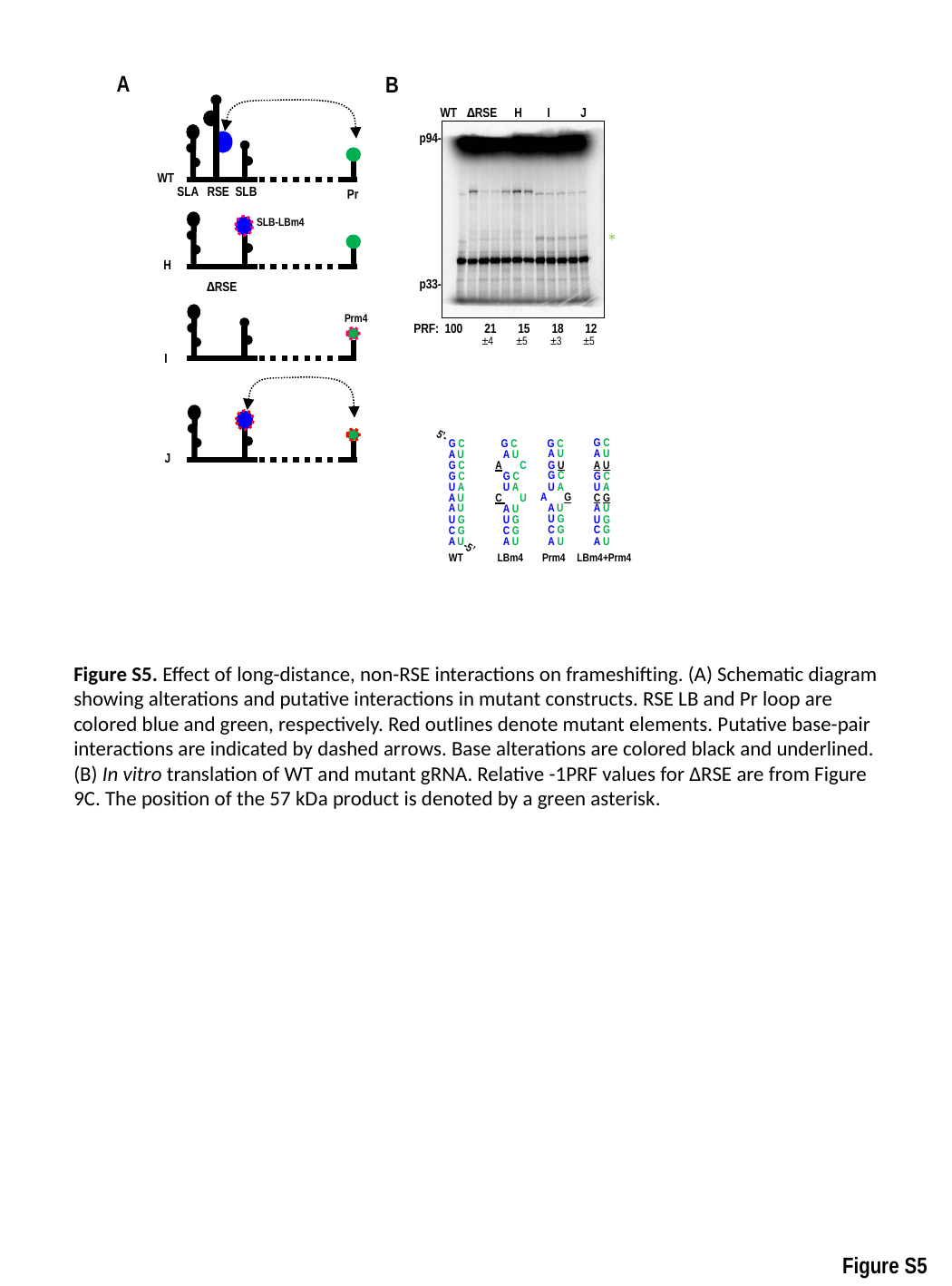

A
B
WT
∆RSE
H
I
J
p94-
p33-
PRF:
100
21
15
18
12
±4
±5
±3
±5
WT
SLA RSE SLB
Pr
SLB-LBm4
*
H
∆RSE
Prm4
I
5’-
G C
G C
 G C
 G C
 A U
 G C
 G C
 U A
 A U
 A U
 U G
 C G
 A U
 A U
 G U
 G C
 U A
 A G
 A U
 U G
 C G
 A U
 A U
 A U
 G C
 U A
 C G
 A U
 U G
 C G
 A U
 A U
 A C
 G C
 U A
 C U
 A U
 U G
 C G
 A U
J
-5’
WT
LBm4
Prm4
LBm4+Prm4
Figure S5. Effect of long-distance, non-RSE interactions on frameshifting. (A) Schematic diagram showing alterations and putative interactions in mutant constructs. RSE LB and Pr loop are colored blue and green, respectively. Red outlines denote mutant elements. Putative base-pair interactions are indicated by dashed arrows. Base alterations are colored black and underlined. (B) In vitro translation of WT and mutant gRNA. Relative -1PRF values for ∆RSE are from Figure 9C. The position of the 57 kDa product is denoted by a green asterisk.
Figure S5

## Slide 7
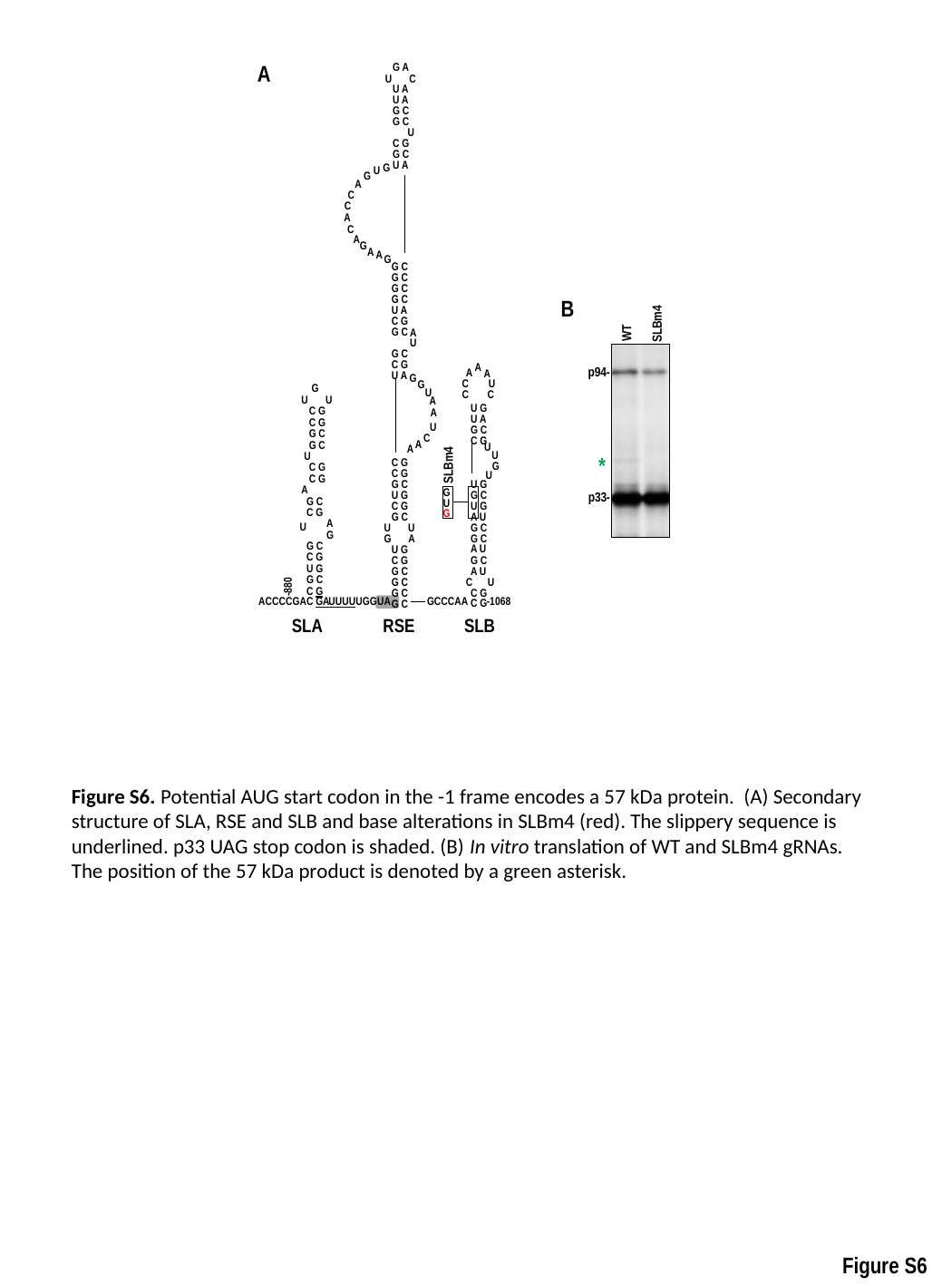

A
 G A
U C
 U A
 U A
 G C
 G C
 U
 C G
 G C
 U A
 G C
 G C
 G C
 G C
 U A
 C G
 G C
 G C
 C G
 U A
 C G
 C G
 G C
 U G
 C G
 G C
 U U
 G A
 U G
 C G
 G C
 G C
 G C
 G C
G
U
G
A
C
C
A
C
A
G
A
A
G
A
U
G
G
U
A
A
U
C
A
A
UUUUUGGUA
A
A
A
C
U
 U G
 U A
 G C
 C G
 U G
 G C
 U G
 A U
 G C
 G C
 A U
 G C
 A U
 C U
 C G
 C G
C
C
U
U
G
U
GCCCAA
 G
 U U
 C G
 C G
 G C
 G C
 U
 C G
 C G
 A
 G C
 C G
 A
 G
 G C
 C G
 U G
 G C
 C G
U
ACCCCGAC GA
B
SLBm4
WT
p94-
SLBm4
*
G
U
G
p33-
-880
-1068
SLA
RSE
SLB
Figure S6. Potential AUG start codon in the -1 frame encodes a 57 kDa protein. (A) Secondary structure of SLA, RSE and SLB and base alterations in SLBm4 (red). The slippery sequence is underlined. p33 UAG stop codon is shaded. (B) In vitro translation of WT and SLBm4 gRNAs. The position of the 57 kDa product is denoted by a green asterisk.
Figure S6
